# The 1933 Long Beach Earthquake (California, USA): Ground Motions and Rupture Scenario

**DOI:** 10.1038/s41598-020-66299-w

**Published:** 2020-06-22

**Authors:** S. E. Hough, R. W. Graves

**Affiliations:** United States Geological Survey, 525S. Wilson Avenue, Pasadena, California 91106 USA

**Keywords:** Solid Earth sciences, Seismology

## Abstract

We present a synoptic analysis of the ground motions from the 11 March 1933 Mw 6.4 Long Beach, California, earthquake, the largest known earthquake within the central Los Angeles Basin region. Our inferred shaking intensity pattern supports the association of the earthquake with the Newport-Inglewood fault; it further illuminates the concentration of severe damage in the town of Compton, where accounts suggest vertical ground motions exceeding 1 *g*. We use a broadband simulation approach to develop a rupture scenario for this earthquake, informed by the damage distribution. The predicted shaking for a 25-km-long fault matches the intensity distribution, with an indication that non-linear site response on soft sediments in some near-field regions was stronger than predicted using a simple model to account for non-linearity. Our results suggest that the concentration of damage near Compton can be explained by a combination of local site amplification, source-controlled directivity, and three-dimensional basin effects whereby energy was channeled towards the deepest part of the Los Angeles Basin.

## Introduction

The 11 March 1933 Mw 6.4 Long Beach, California, earthquake was a landmark event. It remains the largest documented event in the central Los Angeles Basin region, causing widespread damage, and about 120 fatalities^1^. It also occurred at a pivotal point historically, ending a vocal debate about seismic hazard in the region^2^, and providing the impetus for improved building codes^3^. Additionally, the earthquake was not only large enough to be recorded teleseismically, but also occurred soon after the start of the instrumental era in southern California. Strong motion instruments were first installed in the area in 1932^4^, and as of 1933 the fledgling local network comprised 7 stations^5,6^.

**Figure 1:**
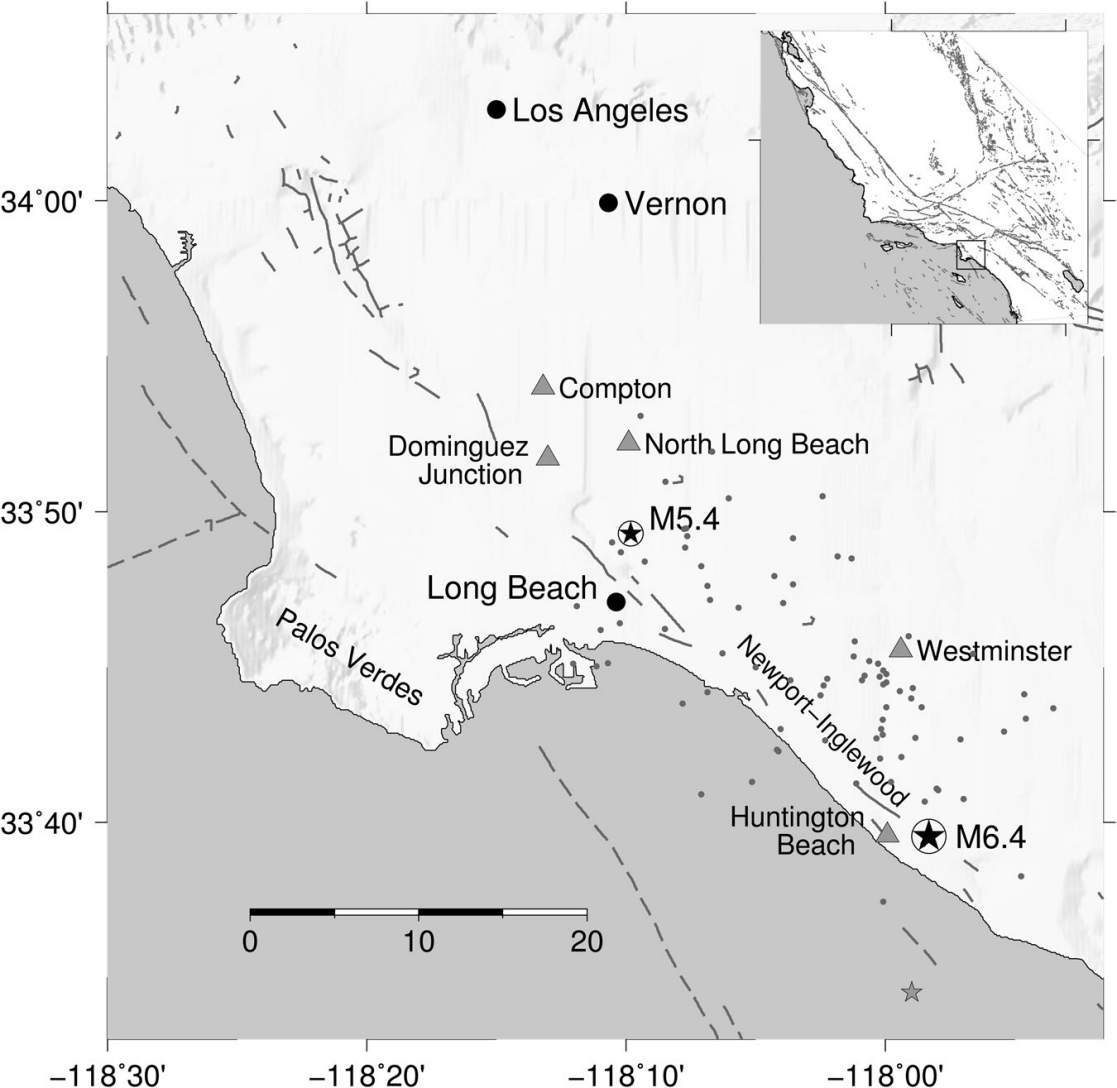
Map of the Los Angeles Basin, including epicenters of the 11 March 1933 M6.4 mainshock (large circled star^9^), 2 October 1933 M5.4 aftershock (small circled star; current catalog location), original (offshore) epicenter location from Wood *et al*.^1^ (gray star), key towns (gray triangles), strong motion stations (black dots), and relocated aftershocks (small gray dots)^9^. Gray lines are mapped and inferred (dashed lines) fault segments, including the Newport-Inglewood fault zone (labeled). Inset map shows location of Los Angeles Basin within California; in this study, we reconsider all available macroseismic data within the region shown.

The Long Beach earthquake did not produce surface rupture^1^. Initial investigations estimated an epicenter about 5 km offshore of Huntington Beach (Fig. 1), with unilateral propagation to the northwest along the Newport-Inglewood, strike-slip fault (NIF)^1^. Some early studies suggested that the event had been centered closer to a concentration of severe damage in the town of Compton (Fig. 1)^7^. Subsequent investigations, however, supported the association with the NIF^8–11^. In particular, Hauksson and Gross^9^ presented a comprehensive analysis of weak motion and teleseismic data, estimating source properties of the mainshock and larger aftershocks. Their analysis supported the conventional interpretation that the earthquake ruptured the NIF^1,10^; with a seismic moment of 5 × 10^25^ dyne-cm (moment magnitude, *M*_*w*_ 6.43), and rupture extending at least 13–16 km unilaterally to the northwest (Fig. 1). The modern catalog estimate is *M*_*w*_ 6.4^6^. Hauksson and Gross^9^ concluded that the source time function and distribution of aftershocks suggest that the mainshock had two significant subevents, although the subevents are not well resolved. Their focal mechanism is pure dextral strike-slip with a NW-SE strike, matching expectations for the NIF. Aftershock locations are distributed over a ~25-km swath to the northwest and about 18 km northeast from the surface expression of the NIF (Fig. 1). However, due (mostly) to irreducible clock errors^6^, even the relocated hypocenters are not well constrained, with especially limited depth resolution^9^.

In this study we revisit the ground motions and rupture scenario of the 1933 Long Beach earthquake. Although extensive macroseismic data was collected by government agencies and special surveys (see Supplemental Material), a comprehensive investigation of ground motions was never undertaken. Key questions, including the concentration of damage in the Compton area, have never been addressed beyond cursory explanations involving local site response. We first reconsider extensive damage reports and other macroseismic information^12,13^, as well as limited strong motion data, to map ground motions caused by the event. We then use a broadband computational simulation approach^14^ to explore possible rupture scenarios for the earthquake, informed by the first-order features of the observed distribution of shaking.

## Seismotectonic Setting

The seismotectonic structures of the Los Angeles region have been investigated in considerable detail. Within the Los Angeles depositional basin, northwest-trending groups of faulted anticlines were initially recognized as oil-producing trends in the early 20^th^ century^15–17^. The Newport-Inglewood trend was first recognized as a fault zone (Fig. 1) based on evidence of right-lateral displacement and a northwest-southeastorientation parallel to other major right-lateral faults in California^17,18^. Yeats^17^ concluded that right-lateral shear within the Los Angeles Basin has been localized on the Newport-Inglewood zone since the Late Pliocene. Hauksson^19^ showed that the Newport-Inglewood fault zone is associated with a diffuse trend of seismicity, although adjacent to the city of Long Beach, seismicity is offset 4–5 km to the east. Boles *et al*.^20^ concluded that the zone is a deeply rooted, long-lived tectonic feature. While the NIF is generally interpreted as a major right-lateral structure, and the causative fault of both the 1920 Mw~5.0 Inglewood earthquake^21^ and the 1933 Long Beach earthquake^1^, given the complex nature of the zone and the absence of clear surface geomorphic expression, the structure of the NIF has remained enigmatic. A detailed examination of the finite-fault rupture process of the 1933 earthquake has also never been explored.

## Ground Motions

### Instrumental data

From the available strong motion records^22^, peak ground acceleration (PGA) values of 2.8 m/s^2^, 2.2 m/s^2^, and 0.6 m/s^2^ are estimated in Long Beach, Vernon, and Los Angeles, respectively (Fig. S1a; see Supplemental Material). The horizontal records from Long Beach were clipped, so the estimated PGA at Long Beach is a lower bound; given that the instrument would have saturated at PGA values of 2–3 m/s^2^ ^22^, the recorded value at Vernon might be a lower bound as well. Using a published intensity-PGA relation^23^, the recorded PGA values correspond to decimal intensity values of 8.4, 7.6, and 6.0 at Long Beach, Vernon, and Los Angeles, respectively. Limitations notwithstanding, these estimates provide an independent comparison for intensities estimated directly from macroseismic information. Moreover, the record from Long Beach further suggests at least two distinct sub-events (see Supplemental Material).

### Macroseismic observations

Extensive macroseismic data were collected soon after the Long Beach earthquake (see Supplemental Material)^24,25^; these data can be used to determine intensity values using the coeval Modified Mercalli Intensity (MMI) scale^26^ that has been shown to be generally equivalent to the recent EMS-98^27^. Although for many locations there is not enough information to assess the statistical incidence of damage to various building types, the detailed guidelines developed for the European Mediterranean Seismic scale (EMS-98)^28^ are still useful to inform intensity assignments. In some cases, detailed reports do mention the statistical incidence of damage, making application of the EMS-98 guidelines more straightforward.

**Figure 2:**
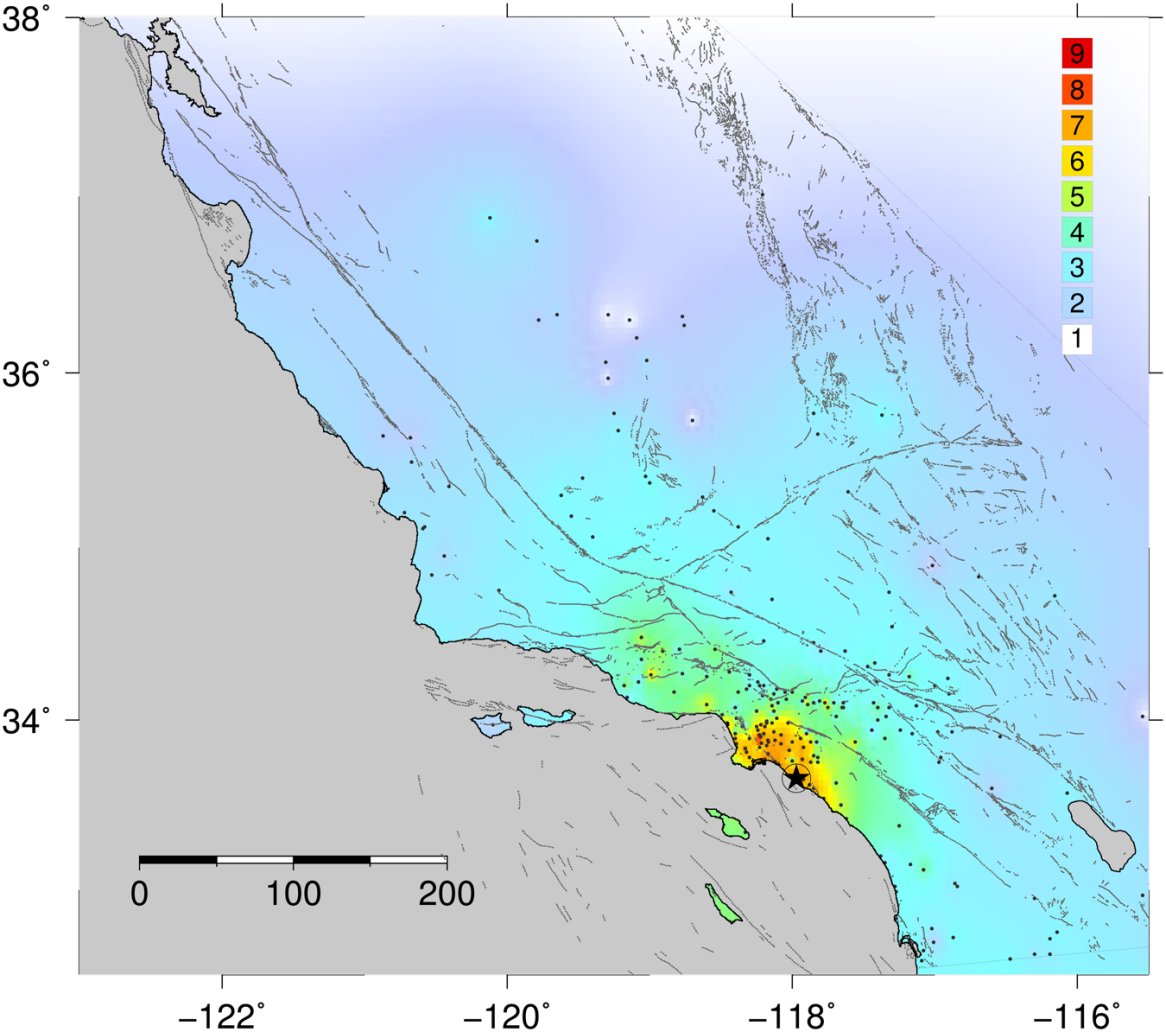
Interpolated overall intensity distribution (city values; this study [see Supplemental Material]). Intensity field (color scale indicated) is interpolated between control points (small dots).

We review the original MMI assignments^25^, which are generally consistent with the values that we assigned based on the documented effects (see Supplemental Material). We do not assign numerical intensities at locations where only environmental effects, such as liquefaction or ground failure are described, as these effects are now recognized to be potentially unreliable indicators of shaking intensity^29^. For locations for which Maher^25^ included multiple accounts and MMI assignments, we average the individual intensity values to obtain an average value for the location (Fig. 2). Our final reviewed set of estimated intensity values agree well with available instrumental intensity estimates discussed above, apart from the Long Beach station, where the observed PGA value was a lower bound.

We additionally consider other sources of macroseismic data, including newspaper reports, detailed reports, and photographs^13,30–32^. (Supplemental Material). A search of archival sources (primarily newspaper accounts) reveals reports from 21 towns not included in the initial report^25^. For photographs and accounts from specified locations, we again use EMS-98 guidelines^28^ to inform assessment of intensity. These point-wise assignments, denoted MMI_PW_, are expected to differ from the city values, which are based on summaries of overall effects, presumably averaging over a range of values within a spatial footprint^33,34^. Given the expectation that especially dramatic instances of damage are more likely to be documented, MMI_PW_ values will be generally higher than city-based values.

**Figure 3:**
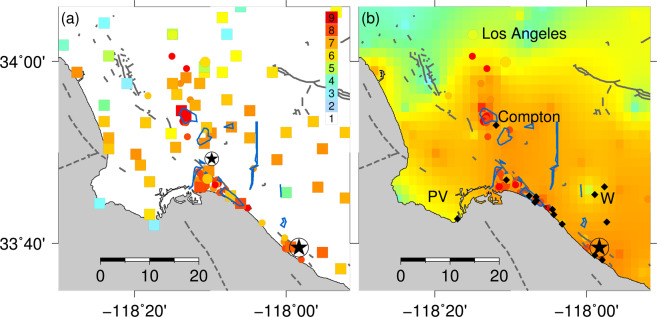
(**a**) (left) Intensity assignments for cities and towns (filled squares) and for individual structures (filled small circles); instrumental intensity values from recorded PGA values also shown (larger circles). Regions outlined in blue correspond to zones with concentrations of broken underground pipes^13^. Epicenters of mainshock and 2 October 1933 aftershock shown (large and small circled star, respectively). Mapped faults are indicated with thick gray lines. (**b**) (right) Same data as shown in 3a, but interpolated intensity field determined from intensity values for cities and towns. Superimposed small filled circles indicate intensities estimated for individual structures; larger filled circles indicate instrumental intensities black diamonds indicate locations where liquefaction or ground failure were documented. W and PV indicate locations of Westminster and the Palos Verdes peninsula, respectively (also see Fig. 1).

To highlight first-order patterns in the spatial distribution, in Fig. 3b we use a linear interpolation scheme to generate a background shaking map using only city-based values, on which we superimpose MMI_PW_ values. Although interpolation can be misleading in areas where there is no information, few areas were entirely devoid of structures by 1933, and, due to the aforementioned tendency for dramatic damage to be recorded, localized instances of significant building damage are unlikely to have been missed. It is more possible, however, for relatively modest effects (MMI ≤ ~5) to have gone unreported in sparsely populated areas, for example along the initial (inferred) ~10-km of the rupture.

Available instrumental data support our estimated intensity values at Vernon and Los Angeles (Fig. 3b). The estimate at Long Beach is lower (decimal intensity 7.4 versus roughly 8.5), but as noted, the only reliable record is from the vertical component, and the horizontal records were clipped.

As discussed by earlier and more recent studies^1,13^, the most severe damage was concentrated in Long Beach and Compton (Fig. 3b). Damage in Compton was especially severe; the proportion of damaged structures was highest in this location, with the most dramatic instances of damage^1^. Four accounts suggest high accelerations in some parts of Compton (see Supplemental Material; Table S2). One compelling account was from the machine shop at Compton Junior College, where heavy machinery reportedly “shot into the air” and came down “many inches” away from the original position. The account noted, “Proof that they did not slide or walk was evidenced by the fact that oil pools around the feet were undisturbed^17^”. Although not unequivocal, these accounts suggest that parts of Compton experienced vertical accelerations in excess of 1 *g*. An additional MMI_PW_ value is assigned for Dominguez Railroad Junction (Fig. 3a), approximately 4 km south of Compton: although this area was sparsely populated, an isolated power station (a small, squat masonry structure) collapsed completely, suggesting that the zone of severe shaking extended at least several km south of Compton.

Observed damage to underground pipes^12^ provides further constraints on near-field ground motions^13^. Concentrations of broken pipes provides an indication of high strain levels, with estimated peak ground velocities (PGV) upwards of 0.2 m/s^14^, which corresponds to MMI upwards of 7^23^. In the Long Beach area, the areas with the most severe damage generally do not overlap the locations where pipes were broken (Fig. 3), suggesting that the highest strains were associated with low-impedance, water-saturated sediments that experienced a pervasively non-linear response^13^. In effect, the expected association between rupture proximity and shaking intensity will break down where pervasive non-linearity causes deamplification of shaking at frequencies of 1–8 Hz^13,35^, which generally control macroseismic effects^36^.

Although we did not assign numerical intensity values for locations at which only environmental effects are documented, in Table S2 (see Supplemental Material) we include locations at which grossly nonlinear sediment response (liquefaction and/or ground failure) was observed^11,27^. Ground failure was observed at a number of locations along the NIF northwest of the epicenter, where numerical intensities are lacking (Fig. 3b). Liquefaction was also documented at a small number of locations at distances of 12–15 km from the NIF, including in some parts of a rural area near Westminster, where MMI 5 was estimated from extant accounts of shaking effects by both the earlier study^21^ and the authors (see Supplemental Material).

As discussed by Trifunac^13^, there is compelling evidence for pervasive non-linear response in North Long Beach, where damage was less severe in regions that experienced high (inferred) strain. It further appears likely that shaking intensity was low in the Westminster area, where gross non-linear response was documented. Ground motions in Compton, however, suggest both high strain and high shaking intensities. Previous studies have explained the concentration of damage in these areas as a consequence of local amplification due to shallow low-impedance sediments^37^, but no detailed investigation of local site response has been undertaken. Moreover, unlike Long Beach, in central Compton a concentration of broken pipes did coincide with heavy structural damage, with 50% of commercial masonry buildings in the city heavily damaged^30^. As summarized above, detailed accounts suggested accelerations in excess of 1 *g* in this location, in the same area where pipes were broken (Fig. 3). In North Long Beach, southeast of Compton, there was also a concentration of broken pipes^30^ and damage, with many commercial masonry buildings sustaining significant damage^30^. Thus, while early intensity assignments could often be inflated^29,38^, we conclude that macroseismic observations suggest both high ground velocities (upwards of 0.2 m/s) and high peak accelerations in the Compton and North Long Beach areas, with intensities reaching MMI 8–9. We note vertical ground motions exceeding 1 *g* on sediment sites are not unprecedented: values exceeding 0.6 *g* were recorded at seven strong motion stations during the 22 February 2011 Christchurch, New Zealand, earthquake, with values exceeding 1 *g* at three stations located on sediments^39,40^.

**Figure 4:**
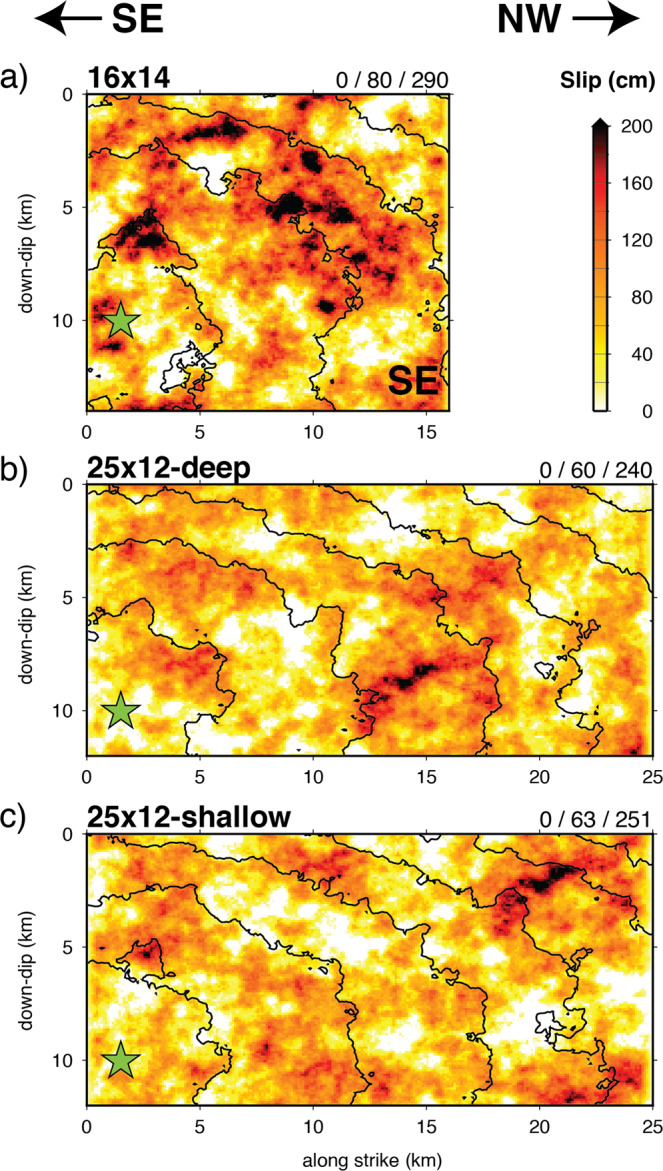
Representative rupture models used in the ground motion simulations: (**a**) 16-km × 14-km fault, (**b**) 25-km × 12-km fault with deep asperity, and (**c**) 25-km × 12-km fault with shallow asperity. Hypocenter is indicated by the green star and black lines are rupture time contours at 2-sec intervals. Triplet of numbers at upper right of each panel are minimum, average, and maximum slip (cm), respectively. For each fault, the top edge is buried at 1 km depth.

In summary, Fig. 3 reveals several first-order features of the intensity distribution: 1) high intensities extending along a roughly north-south line extending from south of Compton towards central Los Angeles; 2) generally higher intensities to the northeast of the Newport-Inglewood fault compared to the southeast; 3) slightly higher intensities at locations to the west bumping up against the Palos Verdes fault (See Fig. 1); 4) lower intensities along the Palos Verdes peninsula; and 5) an overall suggestion of directivity to the northwest. Ground failure and liquefaction was moreover concentrated along the NIF, over a zone extending from a few km south of the epicenter to within the town of Long Beach, 23–25 km northwest of the epicenter.

## Modeling

Well-constrained intensity data can be used to examine possible rupture scenarios using ground motion simulations^41–44^. With these simulations, our goal is not to model the 1933 earthquake in detail, nor to develop an authoritative rupture model for this event, but rather to revisit gross rupture properties, and to explore whether the ground motion distribution is consistent with rupture on the Newport-Inglewood fault. Specifically, we consider whether the shaking distribution is explained better by a 16-km fault, as previously inferred, or a 25-km fault, as delineated by the full length of the aftershock zone. Both rupture lengths are well within the scatter observed in scaling relationships for strike-slip faults^45^. (see Supplemental Material). Given the basic constraints of epicenter and aftershock locations^9^, and the overall distribution of intensities, for both long- and short-rupture models we assume a unilateral rupture towards the northwest following the trace of the NIF. Based on the instrumental results^9^, we fix Mw at 6.45, with average fault strike of 314, dip of 80, and rake of −170. We consider suites of 40 rupture scenarios generated using randomized spatial fields^46^ including fault roughness^47^ for a 16-km long × 14-km wide fault and a 25-km long × 12-km wide fault. Because no surface rupture was observed, the ruptures are all buried at 1-km depth. The 16-km-long rupture is based on the initial aftershock zone^9^, which rapidly extended northward to 25-km within a few hours following the mainshock. The 25-km long ruptures terminate near the location of the Mw5.4 aftershock of 2 October 1933^8^.

We simulate broadband ground motions for these suites of long- and short-rupture models using a hybrid method combining 3-D deterministic and 1-D stochastic approaches^14^. For frequencies less than 2 Hz, we run 3-D finite-difference simulations^48^ using the Southern California Earthquake Center (SCEC) Community Velocity Model – Harvard (CVM-H)^49^ discretized at 40 m spacing and with a minimum shear velocity of 400 m/s. High frequency (>2 Hz) motions are computed in a generic 1-D Los Angeles region velocity structure^1^ modified to have a V_s30_ of 500 m/s. Before summing the low- and high-frequency response, motions are adjusted to the site-specific V_s30_ values^50^ using non-linear site factors^51^. Finally, we use published intensity prediction equations^23^ (see Supplemental Material) to calculate intensities.

**Figure 5:**
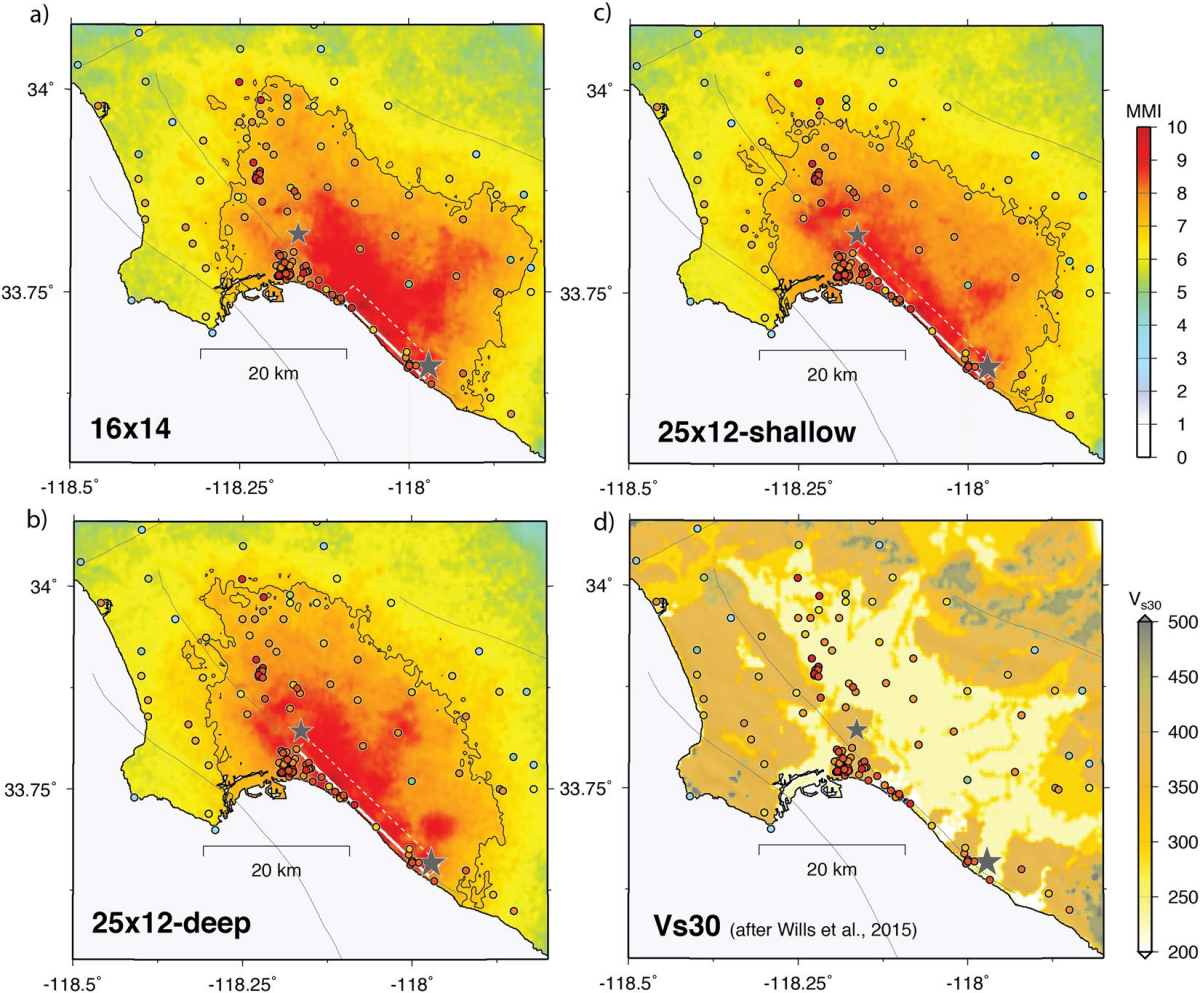
(**a**) Simulated MMI map for the case of 16-km × 14-km fault. MMI is determined from the average of the intensities derived from PGA and PGV of the simulated motion^23^. The black contour denotes MMI of 7. Observed MMI values are plotted as colored circles (see Fig. 3a). Surface projection of the fault is indicated by white rectangle with the mainshock epicenter shown by the large star. Epicenter of M5.4 aftershock indicated by smaller star. (**b**) Same as (**a**) except for 25-km × 12-km fault with deep asperity. (**c**) Same as (**a**) except for 25-km × 12-km fault with shallow asperity. (**d**) Map of V_s30_ values from Wills *et al*.^49^ with observed MMI values superimposed.

For simplicity, we focus here on three representative rupture models: a 16-km rupture with a shallow asperity (Fig. 4a), a 25-km rupture with a deep asperity (Fig. 4b), and a 25-km rupture with a shallow asperity towards the northern end (Fig. 4c). Both the long- and short-fault scenarios are able to match the gross features of the observed intensities, including the generation of larger intensities on the northeast side of the fault due to channeling of energy from the NIF into the deepest part of the central Los Angeles basin (Fig. 5, also see Supplemental Material). We also note that although site conditions are explicitly included in our modeling, observations of high intensity occur on a variety of site types (Fig. 5d).

The 16-km fault scenario concentrates the strongest shaking and highest intensities in the region southeast of Long Beach (Fig. 5a; also see Supplemental Material). Due to the short fault length, this rupture does not produce a strong rupture directivity effect, thus limiting the northwestward extent of high intensities. In contrast, the 25-km rupture scenarios produce strong rupture directivity resulting in a zone of elevated intensities extending northwest of Long Beach that are more consistent with the observations. Although the data are not sufficient to constrain detailed rupture properties, we note that a 25-km fault with a deep asperity (Fig. 5b) generates a larger zone of high intensities (MMI > 7) throughout the central Los Angeles basin compared to a 25-km rupture with shallow asperity (Fig. 5c).

**Figure 6:**
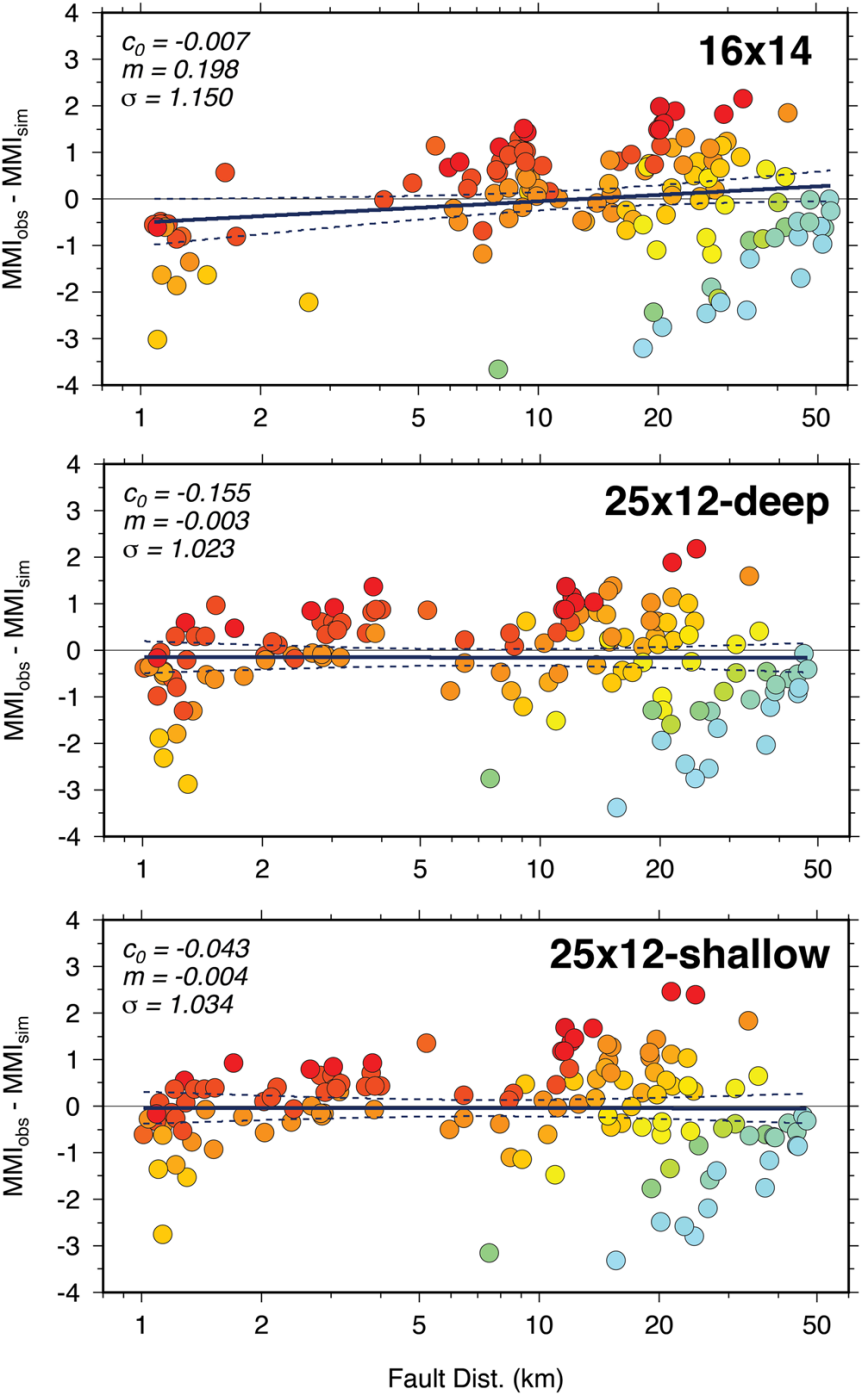
MMI residuals (observed minus simulated) plotted as a function of distance for the three rupture scenarios: 16-km × 14-km case (top), 25-km × 12-km deep asperity case (middle), and 25-km × 12-km shallow asperity case (bottom). MMI is determined from the average of the intensities derived from PGA and PGV of the simulated motion^23^. Residuals are colored according to the observed MMI value. Solid lines are linear regression through the residuals with 95% confidence interval of the mean indicated by dashed lines. The zero intercept (c_0_), slope (m) and standard error (σ) of the regression line is indicated in each panel.

Figure 6 plots intensity residuals as a function of distance to the fault for the three representative scenarios. The 16-km rupture exhibits a significant trend of increasing underprediction with increasing distance, while both 25-km ruptures show little trend with distance. These features seen for the representative ruptures are consistent across the suites of 40 realizations for both the 16-km long and 25-km long ruptures (see Supplement). The 25-km rupture with a shallow asperity rupture has an average misfit slightly lower than the 25-km rupture with a deep asperity rupture, but the latter model better matches (though still under-predicts) the high intensities near Compton.

The observed and simulated intensity values are generally consistent, with the largest misfits occurring for sites very close to the fault or those with relatively low observed intensity at distances over ~20 km. Our simulation model is not able to capture the details of site-specific response that might allow us to better match the observed responses. In particular, the systematic over prediction of intensities in both the near field and other areas where non-linearity was documented, suggests our approach might underestimate the degree of non-linearity that occurred at these sites during the earthquake.

## Discussion and Conclusions

This study presents a first-ever synoptic investigation of ground motions and damage from the 1933 Long Beach, California, earthquake, considering both instrumental and macroseismic data, as well as a modern modeling approach. Using extensive documentation of damage and other effects, including photographs, we conclude that shaking intensities reached MMI 8–9 throughout the near-field region, including within parts of Long Beach and Compton. While data are too sparse to develop a rupture model uniquely, and the models explored in this study are not to be considered authoritative, the first-order. features of the shaking distribution can be generally well explained with a 25-km long, Mw 6.45 NIF rupture. While the distribution of ground motions cannot rule out rupture scenarios involving other faults, they do demonstrate that a different, or more complex, rupture scenario is not required to explain the available ground motion observations. This provides a further measure of support for the conventional interpretation that the earthquake occurred on the NIF. We do find some support, albeit inconclusive, for the previous suggestion of at least two distinct sub-events on the NIF, with the second close to the town of Long Beach. There is an indication that non-linear site response on soft sediments in some near-field regions was stronger than predicted using a simple model to account for non-linearity. Further, our modeling suggests the concentration of damage near Compton may be explained by a combination of source-controlled directivity and three-dimensional basin effects whereby energy was channeled towards the deepest part of the Los Angeles Basin, with local site amplification likely playing a role as well. The results of this study, together with earlier investigations of the 1994 Northridge, California, earthquake^52,53^, suggest that pockets of extreme shaking might be commonly generated by earthquakes in or near complex three-dimensional basins. Such effects will be difficult to predict for future ruptures, but can be explored with increasingly powerful simulation methods such as those used in this study.

## Data and Resources

All accounts analyzed in this study are from published sources; intensity values assigned for this study are provided in the supplemental material.

Strong motion recordings from the 1933 Long Beach earthquake are available from:

Center for Engineering Strong Motion Data (https://www.strongmotioncenter.org/, last accessed 5 July 2018).

SCEC 3D seismic velocity model from: https://github.com/SCECcode/UCVMC, last accessed 31 July 2018.

Fault locations are taken from:

U.S. Geological Survey (and California Geological Survey), 2006, Quaternary fault and fold database for the United States, accessed July 16, 2017, from USGS web site: http://earthquake.usgs.gov/hazards/qfaults, last accessed 28 May 2020.

Latitude/longitude for cities listed in the primary dataset come from the NOAA Earthquake Intensity Database: https://www.ngdc.noaa.gov/hazard/intintro.shtml, last accessed 31 July, 2018.

The large-scale 3D computations were performed using the resources of the Blue Waters sustained-petascale computing project, which is supported by the National Science Foundation (Awards OCI-0725070 and ACI-1238993) and the state of Illinois. Blue Waters is a joint effort of the University of Illinois at Urbana–Champaign and its National Center for Supercomputing Applications. Access to these resources is also part of the “Improving Earthquake Forecasting and Seismic Hazard Analysis Through Extreme-Scale Simulations” allocation made to the Southern California Earthquake Center (SCEC) by the National Science Foundation (Award OAC – 1713792).

Figures were generated using GMT software^54^.

## Supplementary information

Supplemental Material.
