# Supplementary material for: The 1933 Long Beach Earthquake (California, USA): Ground Motions and Rupture Scenario
Source: Sci Rep. 2020 Jun 22;10:10017. doi: 10.1038/s41598-020-66299-w (PMC7308333; doi:10.1038/s41598-020-66299-w)
Supplement: Supplementary file 1 — Supplemental Material. [file 41598_2020_66299_MOESM1_ESM.pdf]

The 1933 Long Beach Earthquake (California, USA): Ground Motions and Rupture Scenario

SUPPLEMENTAL MATERIAL: Strong motion and Macroseismic Data, and Ground Motion Simulations

S.E. Hough and R.W. Graves

U.S. Geological Survey, Pasadena, California

Instrumental Data

The 1933 Long Beach, California, earthquake was recorded by three strong motion instruments that had been installed by the US Coast and Geodetic Survey in 1932; the first true strong ground motion ever recorded in the U.S. (Figure S1a). The original strong motion instruments were torsion pendulum seismometers with a natural period of 0.1s, static magnification of 110, and a sensitivity of 2.5-3 cm/0.1*g*. The record of the vertical component traces from Long Beach could be separated cleanly from the other traces; however, the horizontal component traces overlapped one another, and could not be cleanly separated. Additionally, some peaks on the horizontal components were off-scale.[1]

The fidelity of the strong motion recording from Long Beach is questionable, given the limitations of the instrument and the fact that the horizontal components were both clipped, and impossible to separate. The vertical component recording could, however, be separated cleanly, with turning points distinguishable and on-scale. The fidelity of the vertical component is still questionable, given that ground motions were close to the saturation level of the instrument, but, along with the recordings from Vernon and Los Angeles (Figure S1a), can be analyzed.

To analyze the vertical component from Long Beach, we first integrate the acceleration record. For this calculation, we remove the mean from the acceleration record and apply a 5% Hanning taper before integrating the record. We then apply a high-pass Butterworth filter with a corner frequency of 0.02 Hz, applying a second taper to the filtered time series. The integrated velocity record reveals a fairly simple character. From Figure S1b, we conclude that the instrument triggered on the P wave, capturing the full S wave. We calculate a spectrogram using 2-sec windows with a data slice interval of 1 sec, over the frequency range 0-1 Hz. The

spectrogram (Figure S1b) suggests that there were at least two distinct sub-events, the second larger than the first, with a separation of ~3 seconds in the forward-directivity direction.

Macroseismic Data and Intensities

For the Long Beach earthquake, the United States Coast and Geodetic Survey (USCGS) collected macroseismic information via postcard questionnaire. Summary information was published in an initial USCGS report[2], including a total of 244 locations, most of which are individual towns. Multiple accounts are provided for a few larger cities, presumably from different parts of the city, but without detailed location information. For all accounts, MMI intensities were estimated and included in the initial report[2]. A later USCGS report[1] included generally higher values, which are less consistent with our assessments, and which we discount in favor of the initial assignments (with minor modifications). In most cases, in keeping with modern practice, modifications to initial intensities involve assignments of half units, whereas the original assignments used only whole integer values. In a few cases, we assign values of 2 and 1.5 for reports that indicate shaking was extremely weakly felt, whereas the original assignments were generally 3 for any positive felt report.

In Table S1 we summarize the documented shaking effects of the 11 March 1933 Long Beach, California, earthquake. Initial intensity assignments made by the United States Coast and Geodetic Survey[2] are indicated, as well as the assignments made by the authors. NA indicates "no assignment" made. For the assignments made in this study, NA indicates locations for which no details were found beyond the numerical assignment in the USCGS report[2] or because accounts were deemed insufficient to assign numerical intensities.

In Table S2 we summarize documented effects from specific accounts, including those for which we can estimate a precise latitude and longitude from information provided. Four of the accounts (intensities marked with asterisks) suggest vertical peak accelerations in excess of 1g. Some of these locations cannot be determined precisely, but the location of the Compton Junior College machine shop can be determined within ~200 m (Figure 3a). Numerous other accounts, including the summary report of Martel[3] and a number of photographs available in readily available online repositories, document the severity of damage in the city of Compton.

Seminal work by Ambraseys[4] and many others[5] has established the potential pitfall of overestimated intensity from observed damage to vulnerable masonry structures. Following the Long Beach earthquake, many researchers emphasized that heavy damage was often if not always a consequence of substandard construction[3]. With four credible accounts suggesting vertical PGA in excess of 1*g* in Compton, and severe damage to well-built masonry building, we conclude that shaking severity did reach MMI 9 in this area. By extension, we conclude that intensities in parts of Long Beach, where commercial masonry building damage was close to that in Compton, was also 8-9.

Table S2 also includes locations at which gross non-linear response (either sand blows or ground failure) was documented. Figure S3 shows sand blows in a rural region near the town of Westminster (see Figure 1). An intensity of 5 was estimated for the town of Westminster based on the account of Neumann[1] that store goods were thrown onto the floor. While no account of effects is available from the location shown in Figure S3, the photograph itself reveals no indication of damage to the structures, including windows, a water tank, and a pole-mounted transformer visible to the left of the photo. The absence of reported shaking effects in this as well as a few other locations provides an indirect suggestion that effects were not severe.

Rupture Models

The aftershock data indicate primarily unilateral rupture towards the northwest, extending from an initial length of about 16 km to about 25 km within a few hours after the mainshock [6]. This distribution of aftershocks can be used as a guide to the rupture length of the mainshock, and suggests the representative lengths of 16 km and 25 km that we use in our modeling. There are no observations of surface rupture for this earthquake; however, given the concentration of damage along the Newport-Inglewood fault zone and the inferred strong shaking levels, the rupture likely reached relatively shallow depths. For our modeling, we set the top of rupture for all three scenarios at 1 km depth.

Constraining the down-dip extent of rupture is difficult. While a few of the aftershocks are located at depths up to about 20 km, the vast majority occurred at depths less than about 13 km [6]. Assuming that the seismogenic rupture extended to a depth of about 13 km results in a down-dip width of 12 km for a nearly vertical fault buried at 1 km depth. Magnitude-area scaling relations for strike-slip faulting in active tectonic regions [7,8] predict Mw6.46 for a 25 km long x 12 km-wide fault, which is very close to our assumed magnitude of 6.45. Using these same relations to obtain Mw6.45 for the 16 km long fault would require a rupture depth down to nearly 19 km, which seems extreme given the paucity of aftershocks below 13 km. On the other hand, using a 12 km down-dip width for the 16 km long fault would result in a very high static stress drop event. As a representative compromise, we decide to use an average down-dip width of 14 km for the 16 km long fault (rupture depth of about 15 km). This rupture still has a relatively high static stress drop with an average slip about 30% higher than the 25 km long ruptures.

Another consideration is the distribution of slip within the rupture. Hauksson and Gross[6] suggest the rupture contained two large slip patches based on the apparent clustering of aftershocks and the teleseismically derived source time function, although they caution that this interpretation is not well-constrained given the limited data. Our approach for generating ruptures[9] utilizes random spatial fields, and as such, does not create discrete subevents per se. We calculate ground motions for a total of 40 realizations for both the short- and long-rupture cases, with each rupture realization having a random sampling of slip distribution, average rupture speed, and down-dip fault width (Figures S2a and S2b).

From these realizations, we selected representative ruptures for each fault that had at least two distinct patches of large slip (asperities). This resulted in the ruptures shown in Figure 2a (16-km long x 14-km wide fault) and 2b (25-km long x 12-km wide fault with deep asperity). Both of these ruptures share a common feature of relatively large slip in the region above the hypocenter. The 16-km long rupture has another asperity patch in the shallow portion of the fault about 8 km along strike from the hypocenter. In contrast, the 25-km long rupture with deep asperity has a second large slip patch located in the deeper portion of the fault about 12 km along strike from the hypocenter. Our final representative rupture shown in Figure 2c of the main paper (25-km long x 12-km wide fault with shallow asperity) is simply a variation of that shown in Figure 4b where we have shifted the phase of the slip distribution so that the largest asperity is located at the shallow northwest end of the fault. This was done to test the impact of having large shallow slip very close to the Long Beach area.

Ground Motion Modeling

The region covered by our simulation model is shown in Figure S3. We choose to represent the 3-D seismic velocity structure using SCEC CVM-H[10], which has been shown to provide a better match than other CVMs for observed ~1 Hz waveforms in the LA basin[11]. The distribution of V_{s30} values[12] across our simulation region is also shown in Figure S3. The deep, central portion of the LA basin is aligned with the location of the lowest V_{s30} values. Figure S4 shows cross-sections of shear wave velocity from CVM-H along the three profiles indicated in Figure S3. As seen in these profiles, the Newport-Inglewood fault forms a structural boundary between generally shallower sediments on the southwest and the deeper sediments of the central LA basin to the northeast. Interestingly, the orientation of the velocity contrast across the fault in the upper few kilometers varies along the fault strike. In the south (profile C-C'), somewhat lower velocities are found on the southwest side of the fault, whereas a few kilometers to the north (profile B-B'), the opposite is true.

We use version 18.5 of the Unified Community Velocity Model (UCVM) software[13] to create our 3-D velocity mesh that is used in the deterministic portion ($f < 2$ Hz) of our simulation. Near surface material is modeled using the Ely-Jordan[14] Geotechnical Layer (GTL) with the minimum shear velocity set at 400 m/s. Our staggered-grid finite-difference simulation method requires a minimum of five grid points per wavelength, which results in an upper frequency limit of 2 Hz using a grid spacing of 40 meters. Anelastic attenuation is modeled using a frequency independent Q approximation[15] with $Q_s = 50 * V_s$ (V_s in km/s) and $Q_p = 2 * Q_s$. We save three-component waveforms from the deterministic calculations on a 480 m x 480 m grid of points (27,448 total locations) covering the simulation region.

To generate broadband waveforms, we next simulate three component high-frequency ($f > 2$ Hz) motions at the 27,488 locations using a stochastic approach[16]. The velocity structure

used for the stochastic calculation is represented by a generic 1-D profile having a V_{s30} of 500 m/s (Table S3) and we apply a kappa[17] of 0.045 s for all sites. For both the deterministic and stochastic responses, we apply non-linear, frequency-dependent site response factors[18] to adjust the motions from the reference V_{s30} used in the initial simulation to the site-specific V_{s30} value. Finally, the full broadband (0-20 Hz) time series is obtained by applying a set of matched butterworth filters (crossover at 2 Hz) to the individual deterministic and stochastic responses and then summing in the time domain[16].

Computing MMI from Simulations

For each simulated three-component time series we extract peak ground acceleration (PGA) and peak ground velocity (PGV), and then we use the relations of Worden[15] to compute MMI. The results shown in the main body of the paper use the relation that averages intensities computed from PGA (MMI_A hereafter) and PGV (MMI_V hereafter) to determine MMI. This relation gives slightly smaller errors in fitting observed intensity data compared to using PGV alone[19].

Figure S5a shows the mean of MMI residuals for all 40 realizations of the 16-km long fault plotted as a function of distance. The error bars indicate the standard error of the residuals at each site across the 40 realizations. Figure S5b shows a similar plot for the 25-km long fault case. Comparing Figure S5 with Figure 6 of the main text shows that the trends found for our representative 16-km and 25-km long cases are quite similar to the average trends seen across the 40 realizations for both fault lengths. Additionally, the relatively small error bars indicate that the predicted intensities are generally insensitive to second-order features of the rupture models.

Our results and conclusions remain essentially unchanged if we use MMI_V as illustrated in Figure S6, which shows maps of simulated MMI_V for the three representative rupture scenarios. Comparing the panels in Figure S6 to those in Figure 5 of the main text, we see the same general patterns for each rupture, although some small differences are apparent. The differences can be traced primarily to deterministic features of the 3D seismic velocity structure used for the lower frequency (< 2 Hz) portion of the simulation. Since PGV is more sensitive to the lower frequencies than PGA, these deterministic features are more apparent in MMI_V than MMI. These features include the swath of large intensities extending northward along the deep central LA basin for the 16-km long fault rupture (Figure S6a), as well as the zone of elevated intensities in the Compton area for the 25-km long, deep asperity rupture (Figure S6b). These effects result from channeling and focusing of energy within the 3D velocity structure. Due to computational and knowledge limitations, we are not yet able to deterministically include these features at higher frequencies, and we use a stochastic approach for this bandwidth instead. However, it is likely that similar 3D amplification effects also extend to higher frequencies.[20]

We also note that, for our simulations, using PGA to compute intensity produces systematically lower values (about 0.5 units) compared to the PGV approach, particularly for larger intensities (Figure S7). The reasons that the intensities derived from PGA are different from those derived from PGV in our simulations are likely related to source and ground response features embedded in our simulation approach. First, our kinematic rupture characterization employs depth dependency of rupture speed and peak slip rate (via rise time) such that the rupture speed is decreased and rise time increased in a zone from 0 to 5 km depth. This has the effect of reducing the radiation of higher frequency energy from the shallow portion of the fault,

consistent with observations from past earthquakes showing that shallow rupturing events generate relatively weak high-frequency ground motions compared with deeper ruptures[21,22,23].

Second, when the ground motions are large, non-linear soil response has a much stronger impact on the higher-frequencies (~PGA) compared to the lower frequencies (~PGV). To illustrate the impact of non-linear response on the computed intensities, Figure S8 compares intensities determined for the 25-km long, deep asperity rupture simulation using only linear site response adjustments with those determined using non-linear adjustments. For MMI_A the impact of including non-linearity reduces the computed intensities by 0.2 to 0.6 units for most sites, with a clear trend of stronger reduction for lower V_{s30} sites and larger ground motion. As expected, the impact of non-linearity on MMI_V is much less significant with most sites changing less than 0.2 units. The other rupture scenarios produce similar results to those shown in Figures S7 and S8. As noted in the main text, we suspect that our site adjustment model may underestimate the degree of non-linearity for some sites experiencing very strong motions. Using a site response model with stronger non-linear effects would increase the trends shown in Figures S7 and S8 even more. Due to the limited number of observations at very large MMI, it is not clear at present if the trends shown here for MMI_A and MMI_V in our simulations are also present in actual earthquakes.

The Simulated Wave Field

Figure S9 displays a sequence of snapshots from wave field animations for the three representative rupture scenarios. The motions are low-pass filtered at f < 2 Hz to highlight the impacts of the 3-D structure on the propagating wave field. The results for the three scenarios are generally similar, although there are some important differences. At 12 sec (top row), the

direct S waves are arriving at Long Beach. The motions throughout the near fault area are strongest for the 16-km long rupture, and relatively weaker for the 25-km long rupture with shallow asperity (due to the longer rise time and slower rupture speed across the shallow asperity). At 16 sec (2nd row), the direct S-waves are arriving at Compton. All three ruptures show strong rupture directivity to the northwest. Additionally, the impact of the 3-D structure is clearly evident on the propagating wave field as the wave fronts become contorted by the laterally varying velocity structure. The 25-km fault with deep asperity (middle) has the strongest motions arriving at Compton due to a combination of rupture directivity and channeling within the relatively low velocity basin sediments. At 20 sec (3rd row), the direct waves are just beginning to arrive at downtown Los Angeles. Due to the propagation distance (about 30 km from the fault), the direct S-waves are relatively small at Los Angeles. However, a second set of waves can be seen back closer to the source that are relatively strong. These are basin-generated surface waves created by channeling of energy into the relatively low velocity sediments of the central LA basin. At 24 sec (bottom row), these surface waves are now traveling up the central LA basin towards downtown Los Angeles. The largest motions at downtown for the simulations are these later arriving basin-generated surface waves.

The features described above are further illustrated in Figure S10, which shows broadband ground velocity at Long Beach, Compton and downtown Los Angeles for the three simulations. The waveforms at Long Beach are dominated by the direct S-waves and have a relatively short duration of strong shaking with pulse-like motions. The PGVs at Long Beach range from 26 cm/s to 37 cm/s (MMI 7-8). Compton is hit by strong rupture directivity in the direct S-wave arrivals, and also gets some large amplitude later arriving surface waves. The largest motions at Compton are for the 25-km fault with deep asperity case, which generates a

PGV of 57 cm/s, corresponding to MMI 8.4. The motions at downtown Los Angeles are dominated by late-arriving basin-generated surface waves for all three scenarios with the direct S-wave arrivals being relatively small. The PGVs at Los Angeles range from 11 cm/s to 18 cm/s (MMI 6-7). For completeness, we show the broadband ground accelerations for all three representative ruptures at these sites in Figure S11.

References

(1)Neumann, F. United States Earthquakes, U.S. Dept. of Commerce, Coast and Geodetic Survey Serial No. **579**, 82 pp, Washington D.C. (1935).

(2)Maher, T. Abstracts of reports received regarding the earthquake which occurred in Southern California on March 10, 1933, 56 pp. U.S. Coast and Geodetic Survey, San Francisco (1933).

(3)Martel, R.R. A report on earthquake damage to type III buildings in Long Beach. Earthquake investigations in California 1934-1935, Special Publication, US Dept. of Commerce, Coast and Geodetic Survey, 143-162 (1936).

(4)Ambraseys, N.N. Notes on historical seismicity. *Bull. Seismol. Soc. Am*. **73:6**, 1917-1920 (1983).

(5)O'Rourke, T.D. & Palmer, M.C. Earthquake performance of gas transmission pipelines. *Earthquake Spectra*, **12:3**, 493-527 (1996).

(6)Hauksson, E. & Gross, S. Source parameters of the 1933 Long Beach earthquake, *Bull. Seism. Soc. Am*. **81:1**, 81-98 (1991).

(7)Leonard, M. Self-consistent earthquake fault-scaling relations: Update and extension to stable continental strike-slip faults, *Bull. Seism. Soc. Am.*, **104:6,** 2953-2965 (2014).

(8) Hanks, T.C. & Bakun, W.H. M-logA models and other curiosities, *Bull. Seism. Soc. Am.*, **104:5**, 2604-2610 (2014).

(9) Graves, R.W. & Pitarka, A. Broadband ground-motion simulation using a hybrid approach. *Bull. Seisl. Soc. Am.*, **100:5A**, 2095-2123 (2010).

(10) Shaw, J. H., Plesch, A. Tape, C. Suess, M.P. Jordan, T.H., Ely, G., Hauksson, E., Tromp, J. Tanimoto, T. Graves, R., Olsen, K., Nicholson, C., Maechling, P.J., Rivero, C., Lovely, P., Brankman, C.M., & Munster, J. Unified Structural Representation of the southern California crust and upper mantle, *Earth and Planet. Sci. Lett.*, **415** 1-15. https://doi.org/10.1016/j.epsl.2015.01.016 (2015).

(11)Taborda, R., Azizzadeh-Roodpish, S., Khoshnevis, N. & Cheng, K. Evaluation of the southern California seismic velocity models through simulation of recorded events, *Geophys. J. Int.*, **205**, 1342–1364, doi: 10.1093/gji/ggw085 (2016).

(12)Wills, C. J., Gutierrez, C.I., Perez, F.G. & Branum, D.M. A Next Generation VS30 Map for California Based on Geology and Topography, *Bull. Seism. Soc. Am.*, **105**, doi: 10.1785/0120150105 (2015).

(13)Small, P., Gill, D., Maechling, P. J., Taborda, R., Callaghan, S., Jordan, T. H., Ely, G. P., Olsen, K. B., & Goulet, C. A. The SCEC Unified Community Velocity Model Software Framework. *Seism. Res. Lett.*, **88:5**. https://doi.org/doi:10.1785/0220170082 (2017).

(14)Ely, G.P., Jordan, T.H., Small, P. & Maechling, P.J. A Vs30-derived near-surface seismic velocity model, AGU Fall Meeting Abstract no. S51A-1907, San Francisco, California, December 13–17 (2010).

(15)Graves, R. W. & Day, S.M. Stability and accuracy analysis of coarse-grain viscoelastic simulations, *Bull. Seism. Soc. Am*., **93**, 283-300 (2003).

(16)Graves, R.W. & Pitarka, A. Broadband ground-motion simulation using a hybrid approach. *Bull. Seismol. Soc. Am.*, **100:5A**, 2095-2123 (2010).

(17)Anderson, J. & S.E. Hough. A model for the shape of the Fourier amplitude spectrum of acceleration at high frequencies, *Bull. Seism. Soc. Am*., **74**, 1969-1993 (1984).

(18) Boore, D. M., Stewart, J.P., Seyhan, E., Atkinson, G.M. NGA-West2 Equations for Predicting PGA, PGV, and 5% Damped PSA for Shallow Crustal Earthquakes, *EQ Spectra*, **30**(3), 1057–1085 (2014).

(19)Worden, C.B., Gerstenberger, M.C., Rhoades, D.A., & Wald, D.J. Probabilistic relationships between ground-motion parameters and modified Mercalli intensity in California. *Bull. Seism. Soc. Am*. **102**, 204–221, doi:10.1785/0120110156 (2012).

(20). Hartzell, S., Cranswick, E., Frankel, A., Carver, D., & Meremote, M. Variability of site response in the Los Angeles urban area, *Bull. Seism. Soc. Am.* **87:6**, 1377-1400 (1997).

(21)Kagawa, T., Irikura, K. & Somerville, P. Differences in ground motion and fault rupture process between surface and buried rupture earthquakes, *Earth Planets Space*, **56**, 3–14 (2004).

(22)Shearer, P. M., Prieto, G.A., & Hauksson, E.. Comprehensive analysis of earthquake source spectra in southern California, *J. Geophys. Res*., **111**, B06303, doi 10.1029/2005JB003979 (2006).

(23)Pitarka, A., Dalguer, L.A., Day, S.M., Somerville, P.G., & Dan, K.. Numerical study of ground-motion differences between buried-rupturing and surface-rupturing earthquakes, *Bull. Seism. Soc. Am.,* **99**, 1521–1537 (2009).

(a) (b)

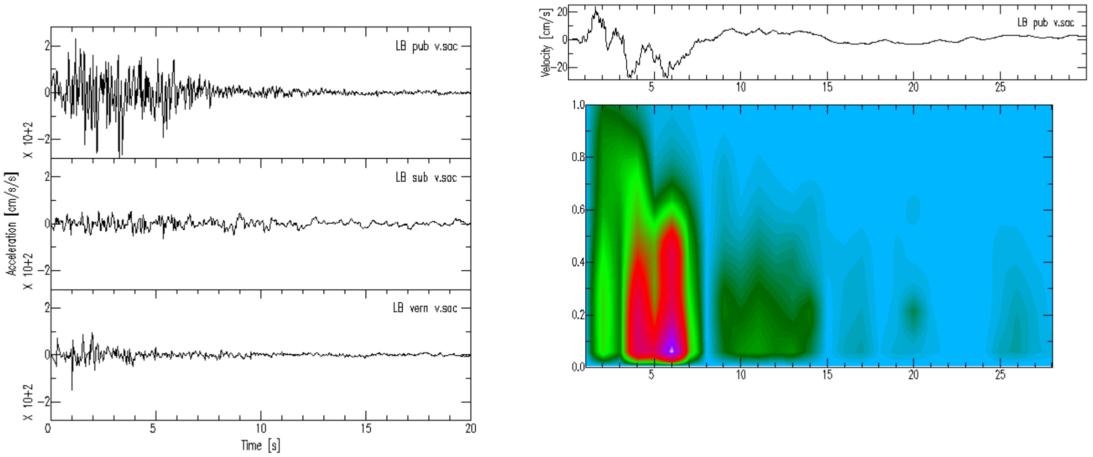

Figure S1. (a) Vertical component of strong ground motion (cm/s/s) recorded at Long Beach (top), Los Angeles (middle), and Vernon (bottom). (b) Vertical component velocity record (integrated from acceleration) from the Long Beach station shown at top; bottom shows spectrogram over a frequency range of 0-1 Hz.

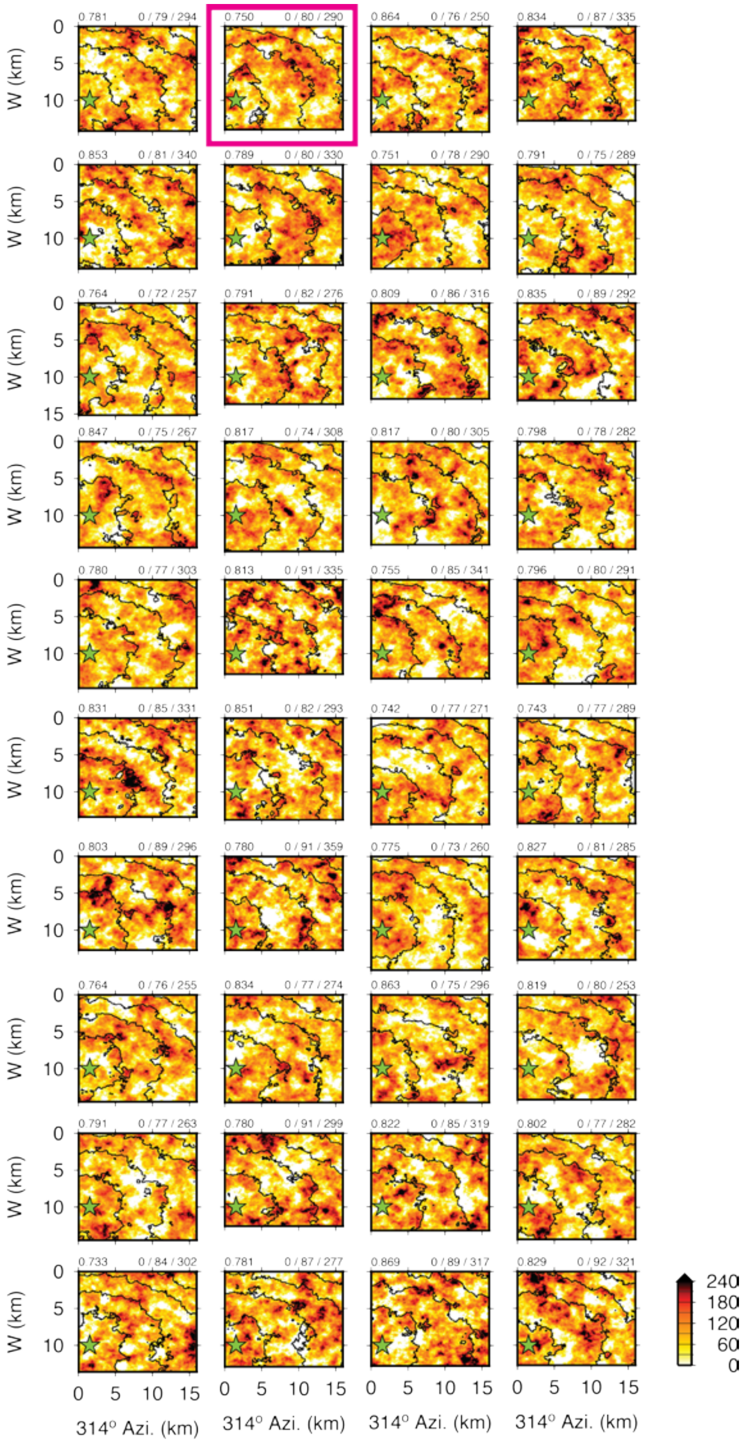

Figure S2a. Suite of 40 realizations of the 16-km long fault case. The realization denoted by the magenta rectangle is the representative case discussed in the main text.

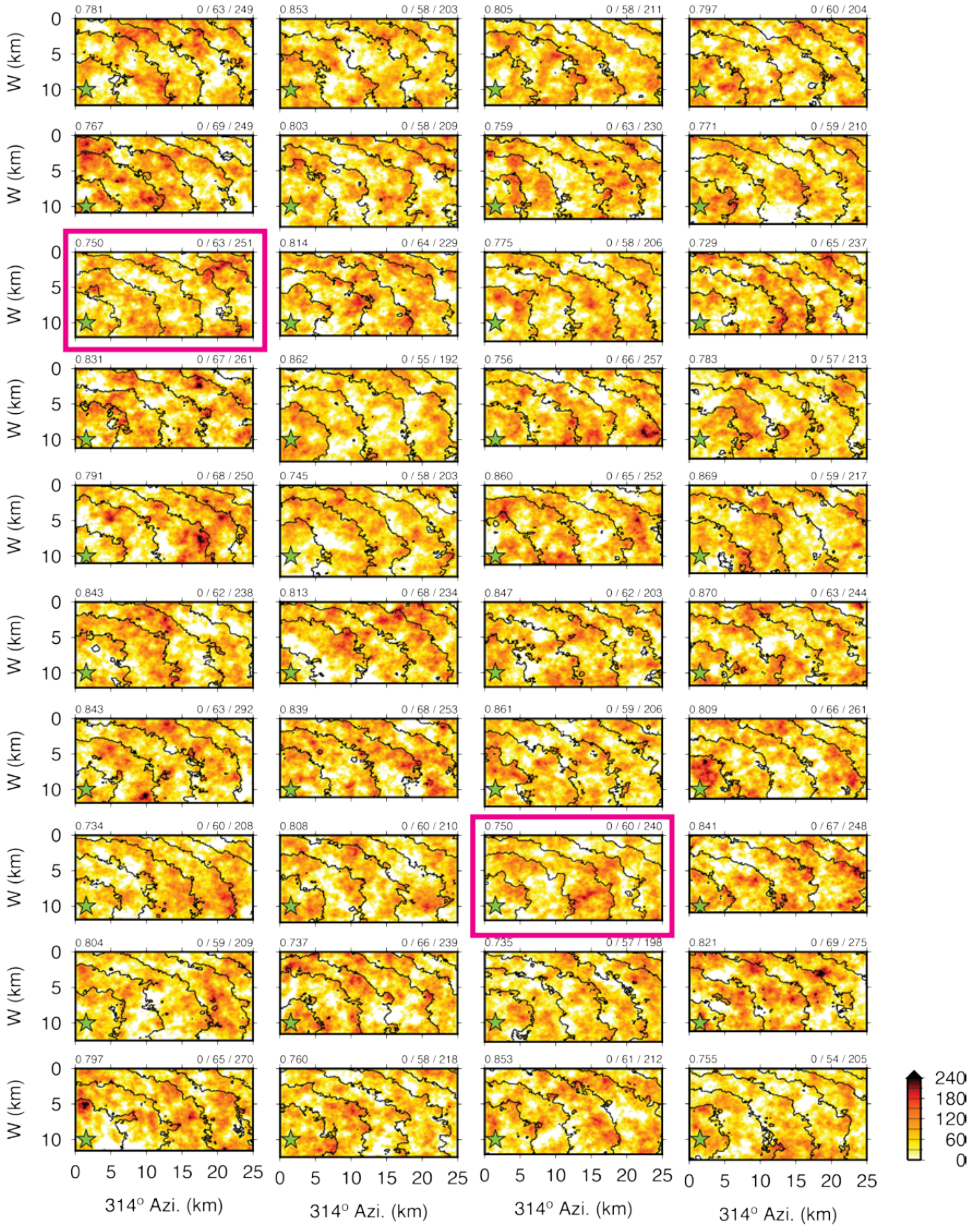

Figure S2b. Suite of 40 realizations of the 25-km long fault case. The realizations denoted by the magenta rectangles are the representative cases discussed in the main text.

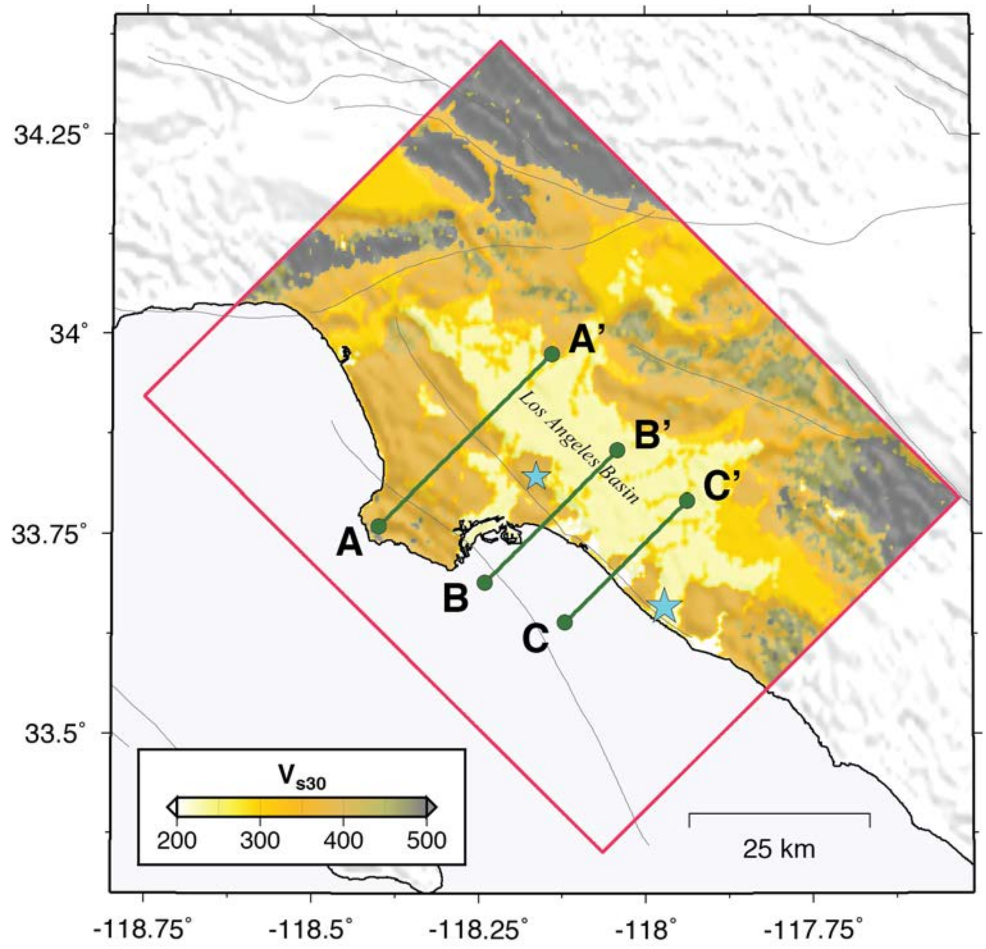

Figure S3. Map showing the region covered by the simulations (magenta rectangle) with color scale indicating V_{s30} values[8]. Large star is mainshock epicenter and small star is M_w5.4 aftershock. Cross-sections of shear wave velocity along indicated profiles are shown in Figure S4.

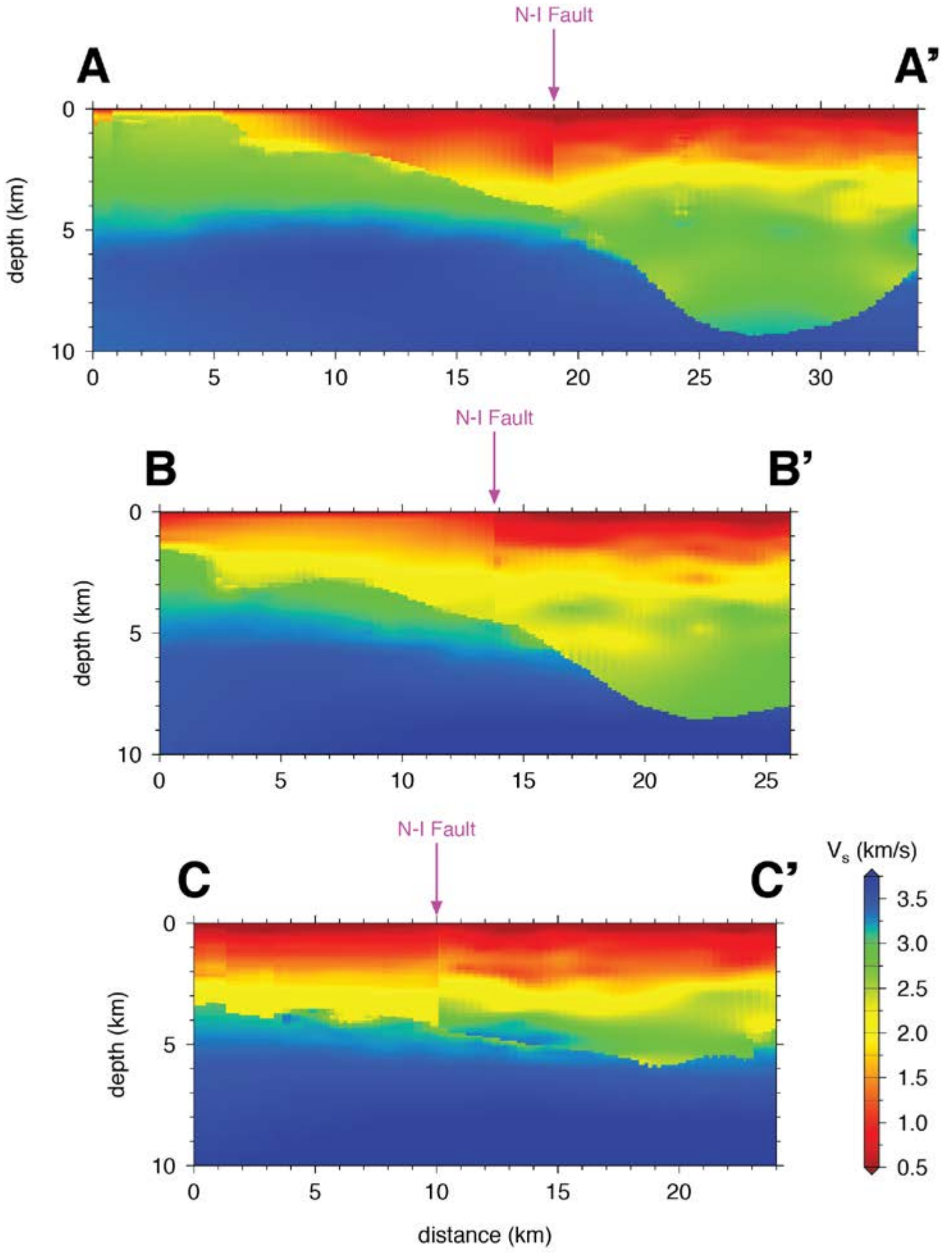

Figure S4. Cross-sections of shear wave velocity from CVM-H[6] along profiles A-A’ (top), B-B’ (middle) and C-C’ (bottom). The location of the Newport-Inglewood (N-I) fault is indicated on each profile.

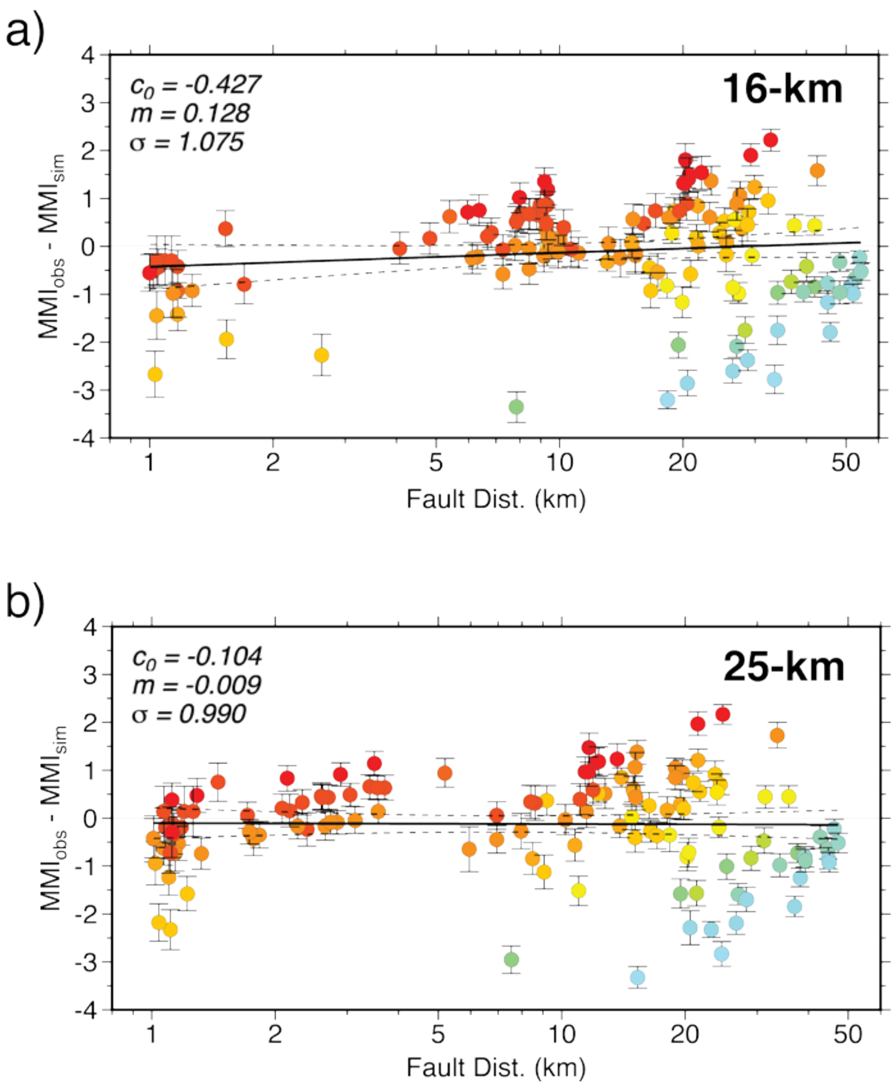

Figure S5. a) Mean MMI residuals plotted as a function of distance for all 40 realizations of the 16-km long fault case. Error bars denote standard error of residuals across the 40 realizations at each site. Mean residuals are colored according to the observed MMI value. Solid lines are linear regression through the mean residuals with 95% confidence interval of the mean indicated by dashed lines. The zero intercept (c_0), slope (m) and standard error (σ) of the regression line is indicated in each panel. b) Same as a) except for 25-km long fault case.

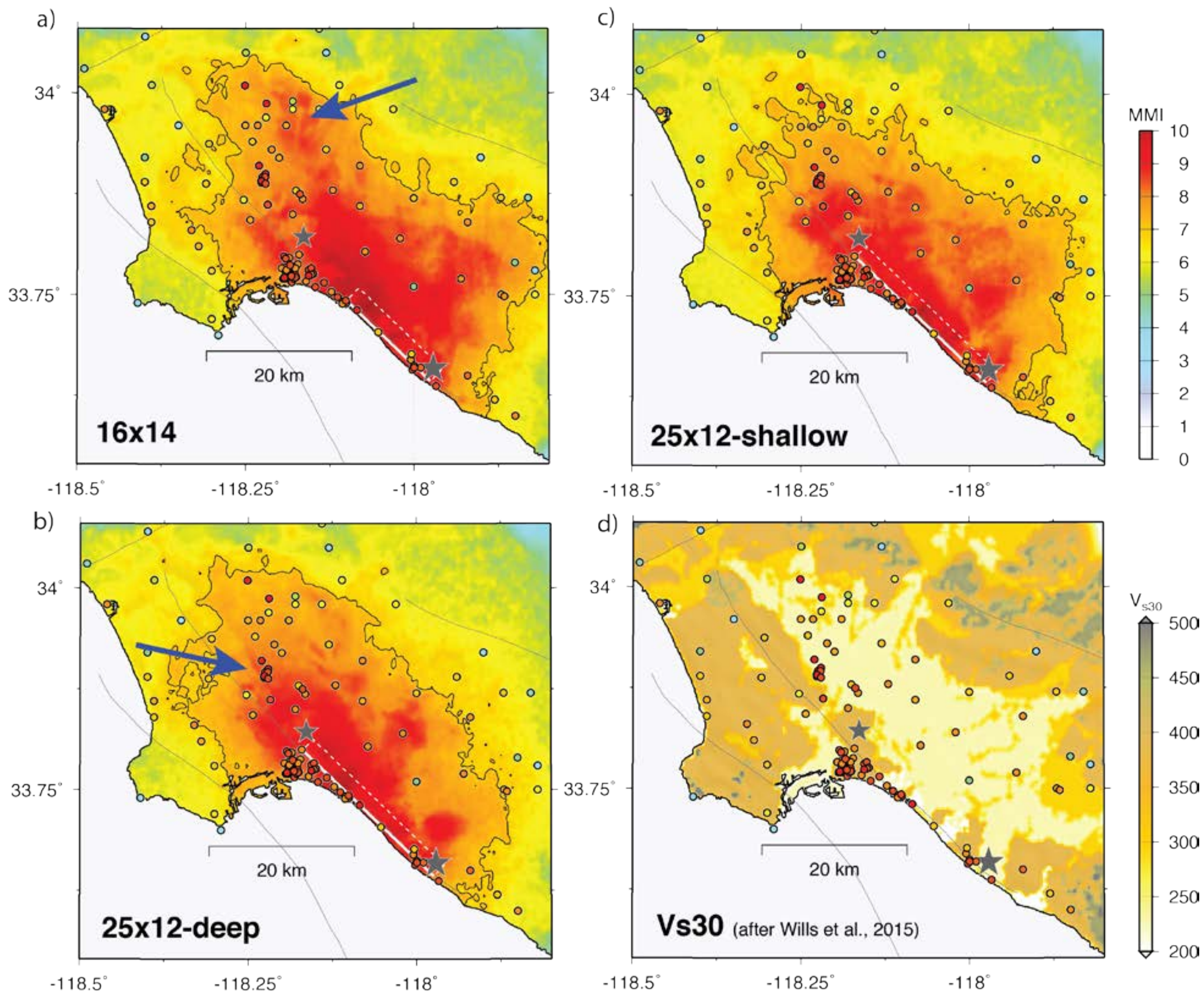

Figure S6. Simulated MMI_V maps for the case of (a) 16-km x 14-km fault, (b) 25-km x 12-km fault with deep asperity and (c) 25-km x 12-km fault with shallow asperity. The black contour denotes MMI_V of 7. Observed MMI values are plotted as colored circles. Surface projection of the fault is indicated by white rectangle with the mainshock epicenter shown by the large star, and epicenter of M5.4 aftershock indicated by smaller star. Arrows on panels (a) and (b) denote deterministic amplification features discussed in the text. (d) Map of V_{s30} values with observed MMI values superimposed (data from Wills et al., 2015).

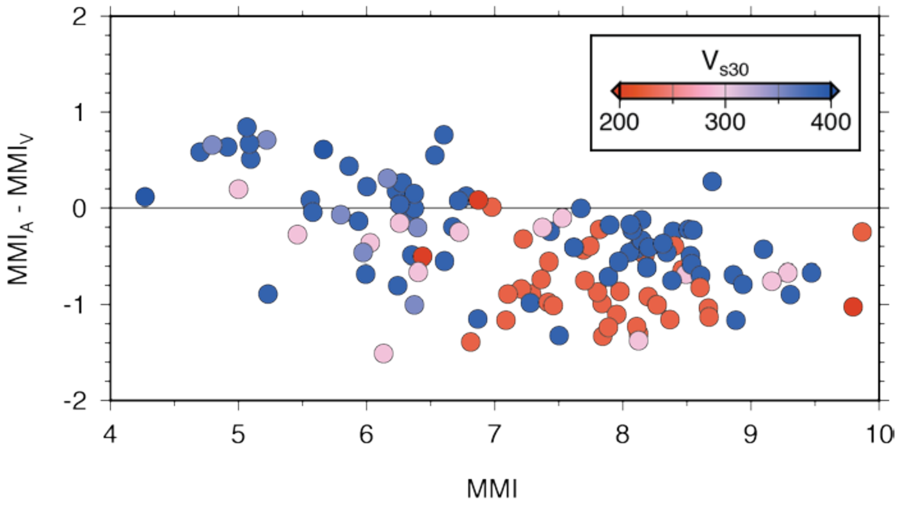

Figure S7. Difference in simulated intensity determined from PGA (MMI_A) versus PGV (MMI_V) plotted as a function of the average of MMI_A and MMI_V (denoted as MMI). For values of MMI above about 6.5, MMI_V is roughly 0.5 units higher than MMI_A. Results here are for the 25-km x 12-km deep asperity case, but are quite similar to that determined for the other scenarios.

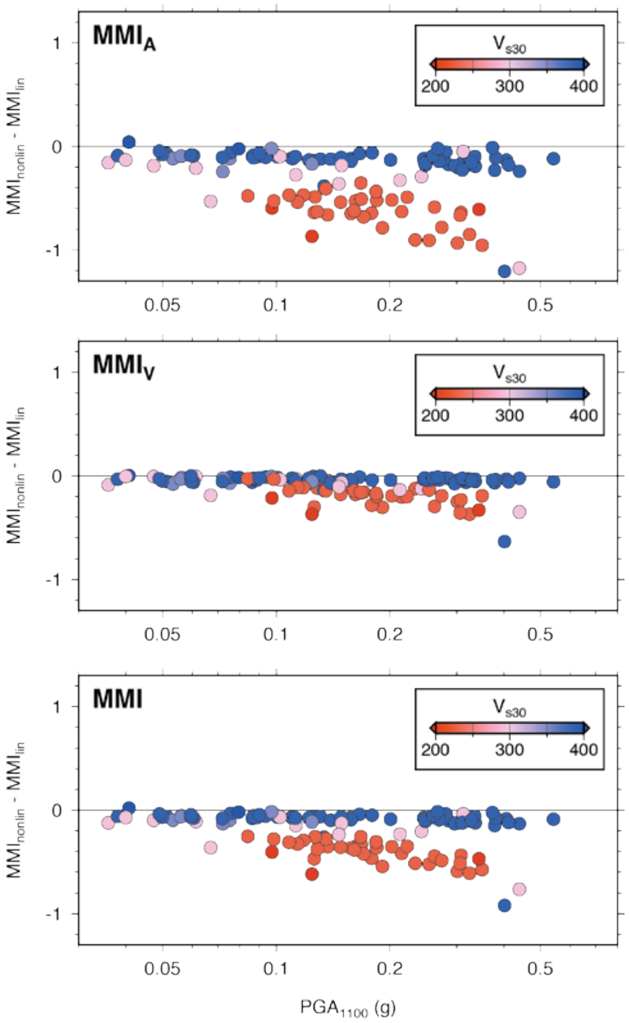

Figure S8. Difference in computed intensities for the 25-km deep asperity simulation using non-linear site response adjustments (MMI_{nonlin}) and those using linear site response adjustments (MMI_{lin}). Values are plotted as a function of PGA_{1100}, which is the simulated PGA value adjusted to a site condition of V_{s30} = 1100 m/s. Color coding of symbols indicates the V_{s30} of the site location. The top panel shows results for MMI_A (MMI computed from PGA), the middle panel shows results for MMI_V (MMI computed from PGV), and the bottom panel shows results for the average of MMI_A and MMI_V.

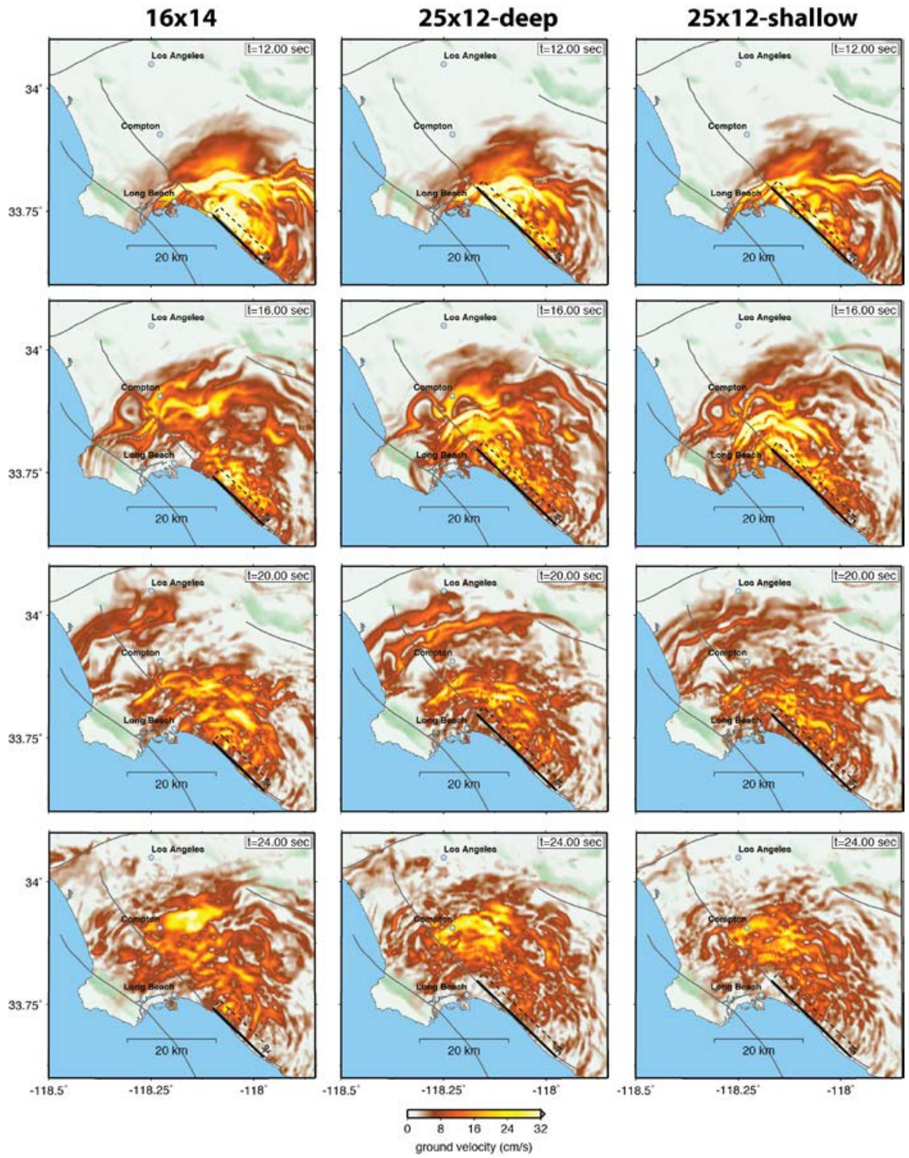

Figure S9. Time slice snapshots of simulated ground velocity along the ground surface for the three rupture scenarios: 16-km case (left column), 25-km deep asperity case (middle column), and 25-km shallow asperity case (right column). Top row is at 12 sec after rupture initiation, 2nd row is at 16 sec, 3rd row is at 20 sec, and bottom row is at 24 sec. Locations for downtown Long Beach, Compton and Los Angeles are indicated on each panel.

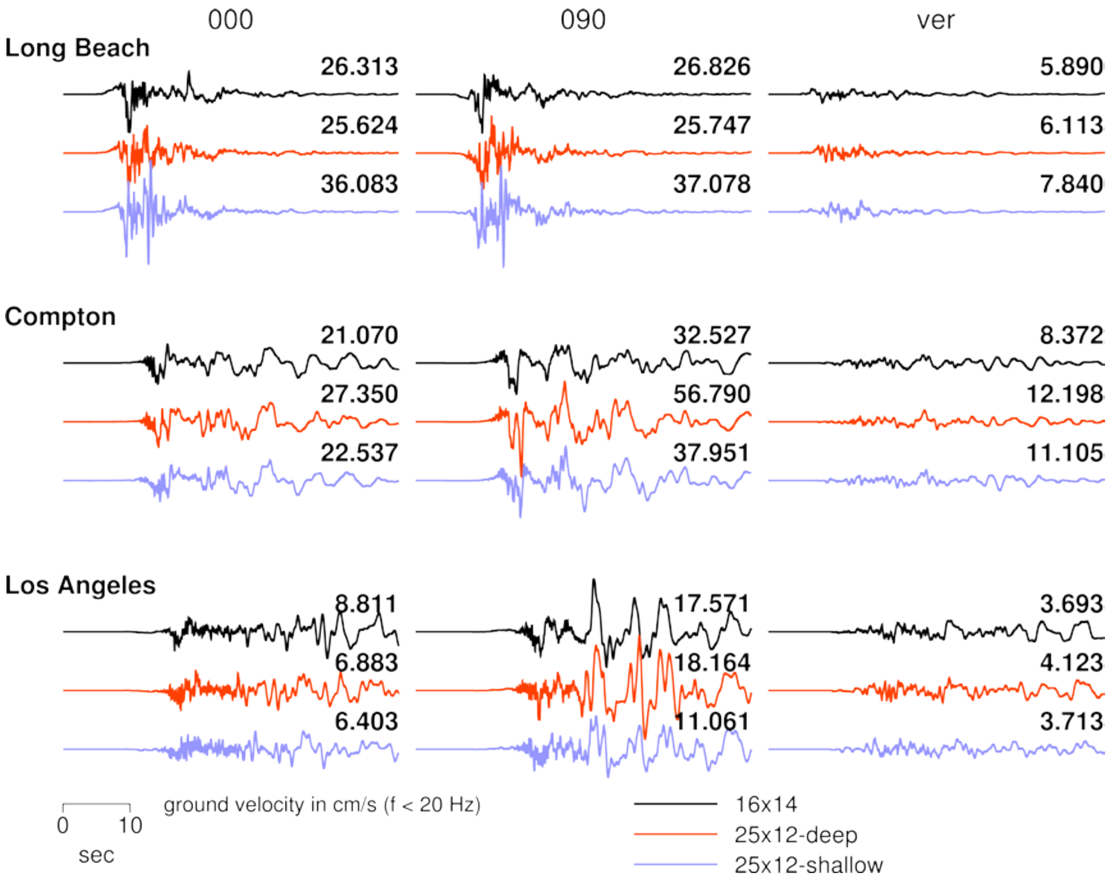

Figure S10. Simulated three-component broadband ground veolcity waveforms for the three rupture scenarios at Long Beach (top panel), Compton (middle panel) and Los Angeles (bottom panel). Number above each trace give the peak value of the simulated motion (in cm/s).

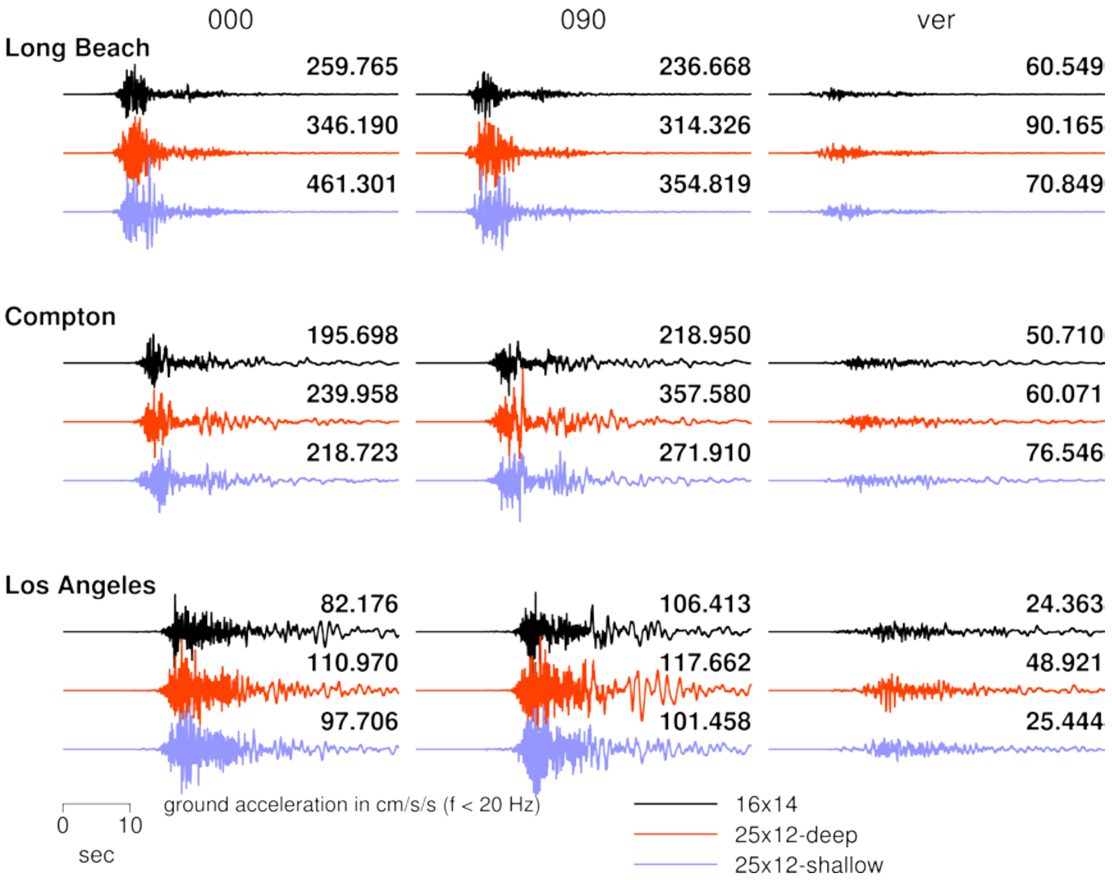

Figure S11. Simulated three-component broadband ground acceleration waveforms for the three rupture scenarios at Long Beach (top panel), Compton (middle panel) and Los Angeles (bottom panel). Number above each trace gives the peak value of the simulated motion (in cm/s/s).

Table S1. Summary of documented macroseismic effects of the 1933 Long Beach earthquake, including intensity assignment from this study (MMI_p) and from Maher (1933) (MMI_o). Effects documented by Maher (1933) are described in that reference, and not reproduced here. Where two different accounts suggest different intensity assignments, both are included. NA indicates no assignment.

MMI_P	MMI_o	City	Lat (°N)	Long (°E)	Effects, reference
1.5	3	Aberdeen	37.01	-118.21	Maher (1933)
4	4	Acton	34.46	-118.2	Maher (1933)
2	3	Adelaide	35.64	-120.87	Maher (1933)
3	NA	Agua Caliente	32.95	-116.3	"Noticed" (San Diego Union, 11 March, page 5)
5	6	Alhambra	34.08	-118.14	"Demolished plate glass in building under construction was only damage" (Los Angeles Times, 13 March, page 15)
4.5	4	Altadena	34.18	-118.11	Maher (1933)
1	NA	Amboy	34.558	-115.74	Not felt (Los Angeles Times, 12 March 1933, page 2)
NA	7	Anacapa Island	34.01	-118.4	Two large landslides, about 1/4 mile apart (Neumann, 1935)
8	8	Anaheim	33.84	-117.92	Roofs of stores caved in, chimneys fell, much brick tumbled into street (San Francisco Chronicle, 11 March)
5	5	Arcadia	34.12	-118.04	Maher (1933)
3	3	Arroyo Grande	35.12	-120.58	Maher (1933)
7.5	7	Artesia	33.86	-118.08	Two men crushed to death in collapse of Scott and Frampton Hall, a large brick structure (Riverside Daily Press, 11 March, page 8); heavy damage to 2-story

					brick buildings in business block (PEER photos)
3	3	Atascadero	35.49	-120.67	Maher (1933)
3	3	Atolia	35.32	-117.61	Maher (1933)
4.5	4	Atwood	33.87	-117.83	Maher (1933)
4.5	5	Avalon	33.34	-118.33	Shaken severely, but no damage; "not even pottery on shelves was broken" (Los Angeles Times, 11 March, pg 15)
3	3	Avila	35.2	-120.72	Felt distinctly by many residents (San Luis Obispo Daily Telegram, 11 March, page 1)
5.5	5	Azusa	34.13	-117.91	"Slight damage" (Neumann, 1935)
4.3	4	Bakersfield	35.37	-119	Average of two assignments (CGS)
NA	8	Balboa Island Bridge	33.63	-117.9	Expansion joints of bridge separated like rifle shocks; tons of dirt from bluffs slid down road (Neumann, 1935)
3	4	Banning	33.93	-116.88	Maher (1933)
1	NA	Barstow	34.896	-117.02	Not felt (Los Angeles Times, 12 March 1933, page 2)
NA	3	Bass Lake	37.33	-119.54	Felt, no further details (Neumann, 1935)
4	4	Beaumont	33.93	-116.98	Maher (1933)
7	7	Bell	33.98	-118.18	Average of two assignments = 6.3 (CGS); Majority of business buildings thrown into street, virtually all plate glass shattered (San Francisco Chronicle, 11 March)
8	8	Bellflower	33.88	-118.12	"Nearly all business buildings razed" (Los Angeles Herald, 11 March)
4	6	Beverly Hills	34.07	-118.4	Maher (1933)

NA	3	Big Creek	37.21	-119.24	Maher (1933)
5	5	Bloomington	34.07	-117.4	Maher (1933)
2	3	Blythe	33.61	-114.6	Maher (1933)
2	3	Blythe	33.61	-114.6	Felt (Los Angeles Times, 12 March 1933, page 2)
NA	3	Bradley	35.86	-120.8	Felt, no further details (Neumann, 1935)
3	3	Brawley	32.97	-115.54	Lighter effects felt in Brawley, Calexico, Oceanside, San Clemente, Santa Barbara, Oxnard, and Santa Paula (San Diego Evening Tribute, 11 March page 8). "Heavy," no damage (San Francisco Chronicle, 11 March 1933)
2		Brawley	32.979	-115.53	"Barely noticeable" (Los Angeles Times, 12 March 1933, page 2)
4	4	Brea	33.92	-117.9	Building shook and car bounced up and down on springs (Ludy, 1933)
4	4	Brown	35.77	-117.85	Maher (1933)
7	7	Buena Park	33.87	-118	Damage estimated at $45,000 (comparable to Garden Grove) (San Diego Union, 12 March, page 2)
5	4	Burbank	34.18	-118.32	Stages shook heavily; no damage described (Washington Post, 12 March)
3.5	3	Buttonwillow	35.4	-119.47	Maher (1933)
4	4	Cajon	34.31	-117.47	Maher (1933)
3	3	Calexico	32.67	-115.49	Felt (San Francisco Chronicle, 11 March)
4	NA	Calexico	32.679	-115.5	"Severe," but caused no damage (Los Angeles Times, 12 March 1933, page 2)
3	3	Caliente	35.29	-118.63	Maher (1933)
NA	4	Camarillo	34.22	-119.04	Assignment, no details (Neumann, 1935)

4.5	NA	Camp Baldy	34.236	-117.66	16-ton boulder hurled from mountainside; lasted half a minute, no damage done (Los Angeles Times, 13 March pg 15).
4	3	Campo	32.59	-116.47	Maher (1933)
4	4	Cardiff by the Sea	33.02	-117.28	Maher (1933)
3	4	Carlsbad	33.16	-117.35	Maher (1933)
4	4	Carriso Gorge	32.71	-116.19	Maher (1933)
3	3	Casmalia	34.84	-120.53	Maher (1933)
3	3	Catalina Island	33.33	-118.33	"Gentle" (Los Angeles Herald, 11 March)
3	3	Cedarpines Park	34.26	-117.32	Maher (1933)
4	4	Charter Oak	34.1	-117.85	Maher (1933)
4.5	4	Chatsworth	34.25	-118.6	Average of two assignments, Maher (1933)
4	4	Chino	34.02	-117.69	Maher (1933)
4	NA	Chula Vista	32.64	-117.08	Sharp shocks; chandeliers swayed; windows rattled (San Diego Union, 10 March, page 1)
3.5	4	Claremont	34.1	-117.71	Maher (1933)
3	NA	Coachella	33.68	-116.17	"Faint" (Los Angeles Times, 12 March 1933, page 2)
4	4	Colton	34.07	-117.32	Maher (1933)
9	9	Compton	33.9	-118.22	Two accounts suggest >1g acceleration (Wood, 1934); Practically all 3000 city structures -- store, office, residences -- either razed or severely damaged (Los Angeles Times 12 March, page 2).
6	4	Corona	33.87	-117.56	Assignment, no details (Neumann, 1935); long crack in one building, bricks fell from chimneys; entire population rushed into streets (Riverside Daily Press, 11 March, page 2).

8	7	Corona del Mar	33.6	-117.85	Maher (1933)
4	NA	Coronado	32.686	-117.18	Sharp shocks; chandeliers swayed; windows rattled.
8.3	8	Costa Mesa	33.65	-117.92	Average of three assignments, Maher (1933).
6	6	Covina	34.09	-117.89	Maher (1933)
5.5	6	Culver City	34.01	-118.39	Shocks were heavy, but little actual damage; small fire started (San Diego Union, 10 March, page 1); plaster cracked and fell at movie studios; cracks in buildings (Washington Post 12 March).
8	8	Cypress	33.82	-118.02	Maher (1933)
3	3	Delano	35.77	-119.25	Maher (1933)
1	NF	Desert Center	33.713	-115.4	Not felt (Los Angeles Times, 12 March 1933, page 2)
4	4	Devore	34.22	-117.41	Maher (1933)
7.5	7	Dominguez Refinery (now Carson)	33.843	-118.243	"Oil refinery badly damaged; dishes broken; otherwise damage slight" (Maher, 1933). (Refinery and town taken to be same location.)
6	7	Dominguez Hills	33.868	-118.253	See above. (Maher, 1933)
8.5	NA	Dominguez Junction	33.862	-118.22	Power station collapsed completely (between Long Beach and Compton) (San Francisco Chronicle, 11 March).
4	3	Dos Cabezas	32.75	-116.14	Maher (1933)
6	7	Downey	33.93	-118.13	Damage to property will not exceed $300; a few broken windows and shattered cornices (Los Angeles Times, 13 March, page 4).
4	5	Eagle Rock	34.14	-118.21	Maher (1933)
3	3	El Centro	32.79	-115.55	Felt, no further details (Neumann, 1935).
4	4	El Mirage	34.4	-117.63	Maher (1933)
4	4	El Modena	33.78	-117.82	Maher (1933)

6	5	El Segundo	33.92	-118.4	"Slight damage" (Neumann, 1935); "severely shaken," but "virtually no damage"; a few chimneys damaged (Los Angeles Times, 13 March, page 15).
5	5	El Toro	33.63	-117.69	Maher (1933)
NA	4	Elsidnore	33.67	-117.33	Assignment, no details (Neumann, 1935).
5.5	5	Escondido	33.12	-117.08	One chimney cracked and some plaster fell; stock fell on floor in stores.
NA	4	Etiwanda	34.13	-117.52	Assignment, no details (Neumann, 1935).
1	3	Exeter	36.3	-119.14	Maher (1933)
3	3	Fairmont	34.74	-118.43	Maher (1933)
5	4	Fallbrook	33.38	-117.25	Maher (1933)
4	4	Fellows	35.18	-119.55	Maher (1933)
5.5	6	Fillmore	34.4	-118.91	Maher (1933)
4	4	Fontana	34.1	-117.43	Maher (1933)
2	3	Fresno	36.75	-119.79	"Some persons say they felt it" (Los Angeles Times, 13 March, page 15).
7	7	Fullerton	33.89	-117.94	Maher (1933)
8	8	Garden Grove	33.77	-117.93	Virtually every building in business district wrecked; one girl killed (San Francisco Chronicle, 11 March page 2)
7	6	Gardena	33.89	-118.3	"Stores damaged" (Neumann, 1935); “Property losses are expected to reach a high figure”; Bank of America building heavily damaged; many store fronts along Palm Avenue down (Los Angeles Times, 12 March 1933, page 15).
3	3	Garnet	33.9	-116.54	Maher (1933)
4.2	6	Glendale	34.2	-118.3	Average of 3 assignments (Maher, 1933).
1	3	Glennville	35.73	-118.7	Maher (1933)

7.5	7	Graham District	33.96	-118.25	Fronts of brick buildings crashed to the street; severe damage; side streets roped off in every direction (Riverside Daily Press, 11 March, page 8).
4	4	Guasti	34.07	-117.64	Maher (1933)
3.3	3	Halcyon	35.11	-120.59	Average of 2 assignments (Maher, 1933).
2	3	Hanford	36.33	-119.65	"Slight" (San Francisco Chronicle, 11 March).
7	6	Harbor City	33.78	-118.3	Chimneys fell, windows broken (Neumann, 1935).
6	NA	Hawthorne	33.92	-118.35	Damage less than $10,000; confined to broken plate glass, firewalls, and cracked outer walls and plaster (Los Angeles Times 11 March page 15).
2	3	Hayfield Camp	33.6	-116.6	Felt, no further details (Neumann, 1935); felt by only a few, and very light (Maher, 1933).
4	4	Helendale	34.74	-117.32	Maher (1933)
3	NA	Hemet	33.75	-116.97	Shock lasted 30-25 seconds, no damage done (Riverside Daily Press, 11 March, page 2).
7.5	7	Hermosa Beach	33.86	-118.39	West wall of a school collapsed; many other buildings damaged (San Diego Union, 10 March 1933, page 1).
4	4	Highgrove	34.02	-117.34	Maher (1933)
3	3	Hipass	32.62	-116.3	Maher (1933)
2	3	Hollister	36.85	-121.4	Maher (1933)
8.3	8	Huntington Beach	33.66	-117.99	Average of two assignments, Maher (1933).

8	7	Huntington Park.	33.97	-118.22	Business district severely shaken; 12 bodies recovered in first four hours (San Francisco Chronicle, 11 March); City Hall and new Christian church considered total loss (San Francisco Chronicle, 12 March).
3	NA	Indio	33.721	-116.22	Felt (Los Angeles Times, 12 March 1933, page 2).
7.5	NA	Inglewood	33.96	-118.35	Over 100 plate glass windows shattered; total damage in excess of $100,000 (Los Angeles Times, 12 March, page 2); City hall tower collapsed; front of Post Office fell in (San Francisco Chronicle 11 March).
3	3	Inyokern	35.65	-117.82	Maher (1933)
4	4	Irwindale	34.11	-117.93	Maher (1933)
3	3	Jacumba	32.62	-116.19	Maher (1933)
4	NA	Jacumba	32.618	-116.19	"Very severe," no damage or other effects reported (Los Angeles Times, 12 March 1933, page 2).
4.5	4	Jamul	32.72	-116.87	Maher (1933)
4	4	Keene Camp	35.21	-118.55	Maher (1933)
NA	4	Kernville	35.75	-118.42	Assignment, no details (Neumann, 1935).
4	4	La Canada	34.2	-118.2	Maher (1933)
4.3	5	La Crescenta	34.22	-118.24	Average of 3 assignments (CGS)
NA	3	La Habra	33.93	-117.95	Felt, no further details (Neumann, 1935)
4	NA	La Jolla	32.83	-117.27	Sharp shocks; chandeliers swayed; windows rattled (San Diego Union, 12 March, page 1)
3.5	4	La Mesa	32.77	-117.02	Maher (1933)
6	6	La Verne	34.1	-117.77	Maher (1933)
4.5	4	La Vina	36.88	-120.12	Maher (1933)

3	NA	Laguna	33.543	-117.79	Felt (Los Angeles Times, 12 March 1933, page 2).
7	7	Laguna Beach	33.54	-117.78	Average of separate 6 and 8 assignments (Maher, 1933).
8	NA	Laguna Beach, downtown	33.543	-117.79	Drug store demolished, walls of 8 downtown buildings caved in; hardly a building escaped some damage (San Francisco Chronicle, 11 March).
6	6	Laguna Bell substation	33.98	-118.14	Cracked plaster and walls, overturned small objects.
5	6	Lake Arrowhead	34.25	-117.18	Maher (1933)
3.7	4	Lancaster	34.7	-118.14	Average of 3 assignments (Maher, 1933)
1	3	Las Vegas	36.17	-115.14	Not felt (Los Angeles Times, 12 March 1933, page 2).
2	3	Lemoore	36.3	-119.78	Maher (1933)
7	6	Lighthipe Station (Hynes)	33.88	-118.17	Cracked plaster, windows, walls, and chimneys; considerable damage to electrical equipment (Neumann, 1935).
2	3	Lindsay	36.2	-119.09	Maher (1933)
3	3	Little Lake	35.94	-117.91	Felt, no further details (Neumann, 1935).
3	4	Llano	34.4	-117.8	Maher (1933)
7.5	7	Lomita	33.81	-118.32	Average of 2 assignments (Maher, 1933).
2	3	Lone Pine	36.61	-118.06	Maher (1933)
8.5	9	Long Beach	33.77	-118.19	Overall building valuation reduced by 9-30% in central Long Beach (Martel, 1934).
NA	4	Los Alamitos	33.78	-118.06	Maher (1933)
5.5	7	Los Angeles	34.05	-118.25	Average of separate assignments (Maher, 1933)
3.5	3	Ludlow	34.72	-116.16	Maher (1933)
7.5	7	Lynwood	33.92	-118.2	One- and two-story brick store buildings badly wrecked; towns seemed almost in ruins; homes not badly damaged (San Diego

					Union, 12 March, page 1)
6.5	NA	Malibu Beach	34.03	-118.78	Movie stars' homes damaged slightly; one chimney toppled (Washington Post, 11 March).
7	7	Manhattan Beach	33.89	-118.4	Plate-glass windows broken, walls cracked, some chimneys down (Neumann, 1935).
4	4	Maricopa	35.06	-119.4	Maher (1933)
5.5	5	Maywood	33.99	-118.18	Windows broken (Neumann, 1935); stocks thrown on floor; considerable losses by businesses and in houses (San Francisco Chronicle, 11 March).
4	3	McFarland	35.67	-119.22	Maher (1933)
4.3	4	McKittrick	35.3	-119.62	Average of two assignments (CGS)
4	4	Mecca	33.57	-116.07	Maher (1933)
3	NA	Mecca	33.572	-116.08	Felt (Los Angeles Times, 12 March 1933, page 2)
4	4	Mentone	34.08	-117.13	Maher (1933)
4	4	Mojave	35.05	-118.17	Maher (1933)
4	4	Monolith	35.12	-118.38	Maher (1933)
5	5	Monrovia	34.16	-118	Dishes broken, bottles knocked from shelves (Neumann, 1935).
6	6	Montebello	34.01	-118.11	City hall damaged (Neumann, 1935)
4	4	Monterey Park	34.05	-118.13	Maher (1933)
4	4	Montrose	34.2	-118.23	Maher (1933)
4	5	Moreno	33.92	-117.16	Maher (1933)
4	3	Muroc	32.9	-117.89	Maher (1933)
7	NA	Naples	33.754	-118.12	Underground pipes broken Trifunac (2003); school damaged; all dishes and bric-

					a-brac broken in home (Rockford Illinois Register Republic, 15 March 1933, page 12).
3	3	Needles	34.84	-114.6	Maher (1933)
4	4	Nestor	32.58	-117.09	Maher (1933)
3	3	Newberry	34.83	-116.69	Maher (1933)
NA	3	Newhall	34.38	-118.52	Felt, no further details (Neumann, 1935)
7	8	Newport Beach	33.62	-117.88	City escaped heavy damage, a few buildings cracked (Maher, 1933)
5.2	5	North Hollywood	34.16	-118.38	Average of three assignments (Maher, 1933)
8	NA	North Long Beach	33.862	-118.18	Underground pipes broken Trifunac (2003); "practically every home damaged," loss of $260,000, mainly to electrical plant (Los Angeles Times, 13 March, page 4)
8	7	Norwalk	33.91	-118.08	Maher (1933)
5	4	Oceanside	33.2	-117.38	"Slight damage" (Los Angeles Herald, 11 March)
4	4	Oildale	35.41	-119.03	Maher (1933)
5.5	5	Ontario	34.07	-117.65	Chimneys and plaster cracked (Neumann, 1935).
5	6	Orange	33.79	-117.85	"Slight damage" (Los Angeles Herald, 11 March).
5.5	6	Oxnard	34.2	-119.18	Maher (1933)
NA	4	Palmdale	34.58	-118.12	Assignment, no details (Neumann, 1935).
4.2	6	Pasadena	34.15	-118.13	Average of three assignments (Maher, 1933)
1.5	3	Paso Robles	35.63	-120.68	Maher (1933)
3	NA	Perris	33.783	-117.23	Felt (Los Angeles Times, 12 March 1933, page 2)
NA	3	Pine Canyon Dam	36.6	-121.64	Felt, no further details (Neumann, 1935)
4	4	Pine Knot	34.24	-116.91	Maher (1933)

1	NA	Pinto Mountains Mining Camp (45 miles east of Mecca)	34.02	-115.54	Not felt at all (Maher, 1933)
4	4	Piru	34.41	-118.79	Maher (1933)
1	3	Pixley	35.97	-119.29	Maher (1933)
7	6	Placentia	33.87	-117.87	Maher (1933)
4	3	Point Conception	34.45	-120.47	Maher (1933)
3.5	3	Point Fermin Light Station	33.7	-118.29	Maher (1933)
3	3	Point Hueneme Light Station	34.13	-119.16	Maher (1933)
5.5	4	Point Loma Light Station	32.67	-117.25	Rated at 5-6 on 10-step scale (San Francisco Chronicle, 12 March).
4	4	Point Vincente Light Station	33.74	-118.41	Maher (1933)
6	6	Pomona	34.07	-117.75	A few chimneys cracked, some plaster fell (Neumann, 1935); Lasted one minute, no damage (San Francisco Chronicle, 11 March)
3	3	Porterville	36.07	-119.02	Maher (1933)
3	3	Prado Dam	33.94	-117.44	Maher (1933)
3	4	Ramona	33.04	-116.86	Maher (1933)
NA	3	Redlands	34.06	-117.18	Felt, no further details (Neumann, 1935)
7	7	Redondo	33.84	-118.39	Five walls jarred loose, fronts of stores damaged, objects broken in stores, two schools damaged (Neumann, 1935)
5		Riverside	34	-117.4	Lasted nearly 30 seconds; windows and dishes rattled, "only a few bricks fell from chimneys in the city" (Riverside Daily Press, 11 March, page 2).
4	5	Riverside	34	-117.4	Average of three assignments (Maher, 1933).

NA	4	Rosamond	34.87	-118.16	Assignment, no details (Neumann, 1935).
NA	4	San Bernardino	34.1	-117.3	Assignment, no details (Neumann, 1935).
5.5	5	San Clemente	33.42	-117.62	"Some buildings suffered slight damage" (Neumann, 1935).
3.4	3	San Diego	33.02	-116.84	Average of 7 separate intensities (Maher, 1933)
4.5	4	San Dimas	34.11	-117.81	Maher (1933)
5	5	San Fernando	34.28	-118.44	Maher (1933)
4.5	4	San Gabriel	34.1	-118.1	Maher (1933)
3.5		San Jacinto	33.78	-116.96	Shock lasted 20-25 seconds, no damage done (Riverside Daily Press, 11 March, page 2).
5.5	5	San Juan Capistrano	33.5	-117.66	"Some buildings suffered slight damage" (Neumann, 1935).
3	3	San Luis Obispo	35.35	-120.41	Felt, no further details (Neumann, 1935); many southern San Luis Obispo County residents felt slight earth shock (San Luis Obispo Daily Telegram, 11 March, page 1).
4	4	San Marcos	33.15	-117.17	Maher (1933)
1.5	3	San Miguel	34.04	-120.38	Felt only slightly (Maher, 1933).
4.5	4	San Nicolas	33.26	-119.5	Assignment, no details (Neumann, 1935); quake intensive enough to cause water to be shaken out of a tumbler within 1/2" of being full, but no damage done (Maher, 1933)
6.5	5	San Pedro	33.72	-118.3	Maher (1933)
7	7	San Pedro	33.72	-118.3	Some buildings badly damaged (San Francisco Chronicle 11 March).

8	8	Santa Ana	33.75	-117.87	Extensive damage to Polytech high school; several cars covered with bricks; Elks club roof collapsed; many buildings "considerably wrecked" (Los Angeles Times, 12 March, page 2)
4	4	Santa Ana River Powerhouse #1	34.2	-117	Maher (1933)
3.7	4	Santa Barbara	34.75	-120.06	Average, three assignments (Maher, 1933).
NA	3	Santa Fe Springs	33.94	-118.06	Felt, no further details (Neumann, 1935).
2.3	3	Santa Maria	34.95	-120.44	Average, two assignments (Maher, 1933).
6.5	6	Santa Monica	34.03	-118.49	Average, two assignments (CGS); City Hall tower collapsed (San Francisco Chronicle, 11 March).
4	5	Santa Paula	34.35	-119.06	China and glassware in store window broken (Neumann, 1935).
1.5	3	Santa Rosa Island	33.97	-120.1	Felt only slightly (Maher, 1933)
5.5	6	Saugus	34.41	-118.54	Maher (1933)
8.5	8	Seal Beach	33.74	-118.1	Underground pipes broken Trifunac (2003); "practically every home damaged," loss of $260,000, mainly to electrical plant (Los Angeles Times, 13 March, page 4).
2	3	Sequoia National Park	36.32	-118.77	Maher (1933)
4	3	Seven Oaks	34.15	-116.9	Maher (1933)
4.5	5	Sierra Madre	34.16	-118.05	Maher (1933)
8	8	Signal Hill	33.8	-118.17	Derricks twisted, tanks and buildings collapsed, oil line broken (Neumann, 1935); broken pipes (Trifunac, 2003).
4.5	6	Simi	34.27	-118.77	Maher (1933)
4	4	Solana Beach	32.99	-117.27	Maher (1933)

7	7	Somis	34.26	-118.99	Chimney thrown from ranch house near Somis (Neumann, 1935).
7.5	8	South Gate	33.96	-118.19	Store fronts collapsed in business district; elevated tanks and buildings collapsed; oil line broken; Grocery store ruined, total damages $600,000 out of $13,000,000 valuation.
6	5	South Pasadena	34.12	-118.15	Maher (1933)
3	3	Springville	34.22	-119.08	Maher (1933)
NA	4	Stanton	33.81	-118	Assignment, no details (Neumann, 1935).
4.5	6	Summit	34.33	-117.42	Maher (1933)
4	4	Sunnymead	33.94	-117.24	Maher (1933)
1	NA	Sweetwater dam	32.69	-117.01	Not noticed (San Diego Union, 11 March, page 5).
4	NA	Tijuana	32.51	117.04	Felt; long bridge "writhed like a snake" (San Diego Union, 11 March, page 5).
2	3	Tipton	36.06	-119.31	Maher (1933)
6	5	Topanga	34.09	-118.6	Maher (1933)
8	7	Torrance	33.83	-118.33	Maher (1933)
5	4	Trinufo	34.16	-118.83	Maher (1933)
4.5	4	Trona	35.76	-117.37	Assignment, no details (Neumann, (1935); card collected by Charles Richter) Felt by all; windows, etc., rattled; house creaked; hanging objects swung; small objects and furnishings moved; vases overturned; trees shaken.
4	4	Tujunga	34.26	-118.3	Maher (1933)
2	3	Tulare	36.27	-118.76	Maher (1933)
6	6	Tustin	33.75	-117.82	A few broken windows and slight dislocation of masonry was the only damage here (Los Angeles Times, 12 March, page 15).
4	4	Upland	34.1	-117.64	Maher (1933)

4.5	4.5	Valyermo	34.45	-117.85	Lasted 30 seconds; house swayed; rather violent but not terrifying; no damage whatever (Levi Nobel letter to Charles Richter).
8	7	Venice	33.98	-118.46	Maher (1933)
6	6	Ventura	34.48	-119.06	Walls of several downtown buildings cracked (San Francisco Chronicle, 11 March)
4	4	Victorville	34.54	-117.3	Maher (1933)
1	3	Visalia	36.33	-119.29	Maher (1933)
3	NA	Volcano Lake, Mexico	32.41	-115.31	Felt (San Francisco Chronicle, 11 March) (Now Cerro Prieto; not present-day Volcano Lake.)
7.5	NA	Walnut Park	33.97	-118.23	Stock strewn on floor in drug store; heavy damage to masonry bank building (Chicago Tribune, 11 March).
3	3	Warner Springs	33.29	-116.63	Felt, no further details (Neumann, 1935).
7.5	8	Watts	33.94	-118.24	Stores fronting on two blocks of 103rd Street were either wrecked or seriously damaged; little damage to residences; interiors of stores were tumbled (Los Angeles Times, 12 March, page 2); Police station collapsed, 3 killed (San Francisco Chronicle, 11 March).
5	5	Westminster	33.76	-117.99	Stock in stores thrown to floor (Neumann, 1935).
3	NA	Westmoreland	33.036	-115.62	Felt (San Francisco Chronicle, 11 March).
NA	3	Wheeler Ridge	35.02	-118.95	Felt, no further details (Neumann, 1935).

6.5	6	Whittier	33.98	-118.03	Plate glass windows shattered; brick cornices thrown down. Elks Temple and numerous other structures damaged; 1/4 of downtown area roped off (San Francisco Chronicle, 11 March).
9	8	Willow Brook	33.91	-118.23	Maher (1933)
7	NA	Wilmington	33.79	-118.26	Damage to City Hall, First National Bank, and Banning High School; damage will not exceed $40,000 (Los Angeles Times, 13 March, pg 15).
5	5	Woodcrest	33.89	-117.35	Small objects moved and overturned (Neumann, 1935).
NA	3	Woodlake	38.15	-121.24	Felt, no further details (Neumann, 1935).
NA	4	Yucaipa	34.04	-117.04	Maher (1933)

References

Maher, T. J. Abstracts of reports received regarding the earthquake which occurred in Southern California on March 10, 1933," 56 pp. U.S. Coast and Geodetic Survey, San Francisco (1933).

Neumann, F. United States Earthquakes, U.S. Dept. of Commerce, Coast and Geodetic Survey Serial No. **579**, 82 pp, Washington D.C. (1935).

Trifunac, M.D. Nonlinear soil response as a natural passive isolation mechanism. The 1933 Long Beach, California, earthquake, *Soil Dynamics and Earthq. Eng.*, **23**, 549-562 (2003).

Table S2. Detailed accounts of damage and other effects. GF = ground failure; N/A = precise location not identified. Intensities with asterisks indicate accounts that suggest vertical ground motions in excess of 1g.

City	Structure	Long. (°E)	Lat (°N)		Effect
Artesia	Business block	N/A	N/A	8	Partial collapse of two-story brick building (Maher, 1933)
Compton	Manual Arts Building	N/A	N/A	9	building leveled (Maher, 1933)
Compton	Stockwell Building (across from Haigh Drug)	-118.225	33.896	9	Total collapse (grade 5 damage); "the center of destruction" (San Diego Evening Tribune, 11 March, page 8)
Compton	House	N/A	N/A	9*	Kitchen stove jumped out of the feet cups, which remained in their original position adhering to the lino, and landed 18" away on the floor (Maher, 1933)
Compton	Junior College	-118.227	33.891	9.5*	Heavy machinery shot into the air and came down many inches away from the first position. Proof that they did not slide or walk was evidenced by the fact that oil pools around the feet were undisturbed (Maher, 1933)
Compton	House (unspecified location)	N/A	N/A	9*	"The floor carpet was certainly below the front feet of the piano before the quake, and 3" away from under after it, and pushed up against the opposite wall" (Maher, 1933)
Compton	Security First National Bank	N/A	N/A	9	Substantial masonry building "demolished" (Associated Press photograph)
Compton	Young Hotel	N/A	N/A	9	"Demolished" (Associated Press photograph)
Compton	House (unspecified location)	N/A	N/A	9	"I was driving through Compton when the earthquake hit. I thought a front wheel had fallen off my car. I saw a two story frame house rocking like a tree in the wind and then I saw it fall." (Daily Telegram, 11 March 1933, Adrian, Michigan, page 1)

Compton	Business district between Alametos and Willowbrook avenues	-118.226	33.896		Business section condemned by Army engineers; "tottering structures" razed. (San Diego Union, 12 March 1933, page 1)
Compton	City Hall; 600 North Alameda St.	-118.22	33.9	9	Collapsed (San Francisco Chronicle, 11 March, page 2); photograph from archives
Compton	Compton Avenue	N/A	N/A	8.5	"Half of the bigger buildings seem to have collapsed" (San Francisco Chronicle, 11 March, page 2)
Compton	Residential area (unspecified location)	N/A	N/A	8.5	"Cottages and apartments of Compton's 3000 residents were twisted on their foundations; many plaster walls caved in." (San Diego Evening Tribune, 11 March, page 8)
Compton	City Jail	-118.221	33.896	8.5	Strongly-built jail resisted the shock but was so damaged seriously the prisoners were moved to a park (San Diego Evening Tribune, 11 March, page 8)
Compton	Liberal Furniture Store	N/A	N/A	8.5	Collapsed (photograph)
Compton	Building and Loan Association	N/A	N/A	8.5	Heavily damaged (photograph, California Institute of Technology archives)
Compton	Between Olive and Cocoa, Willowbrook	-118.226	33.889	9	"Frame dwelling gone" (photograph, California Institute of Technology archives)
Compton	608 S. Acacia St.	-118.226	33.891	9	House in next block moved off of foundation (directly across from Compton Jr. College) (photograph, California Institute of Technology archives)
Compton	Apartment, N. of Compton Ave	N/A	N/A	8.5	Heavy damage, partial collapse (photograph, California Institute of Technology archives)
Compton	Junior High School	33.888	-118.22	8.5	Partial collapse of brick school (photograph, California Institute of Technology archives)

East Long Beach	Methodist church	-118.156	33.781	8.5	Heavily damage to well-constructed masonry building; photograph (Berner, 1981).
East Long Beach	House	N/A	N/A	9*	Marble clock which it took two men to place on the mantel shelf, jumped from its place and was broken in pieces outside the hearth (Maher, 1933)
Huntington Park	House	N/A	N/A	--	Cut glass of various sizes broken within case, but glass doors of cabinet not cracked, nor the cabinet moved in its place (Maher, 1933)
Huntington Park	High School	-118.219	33.987	9	Total collapse of school building (Maher, 1933)
Long Beach	Continental Bakery, Redondo and Anaheim St.	-118.152	33.783	8.5	Partial collapse, 3-story brick bakery building (photograph) ("Most photographed commercial building in town"; Berner 1981)
Long Beach	Jefferson Junior High (7th St. and Euclid Ave.)	-118.149	33.776	9	Partial collapse, two-story school building (Maher, 1933)
Long Beach	Anaheim Blvd	N/A	N/A	8	Partial collapse, 2-story brick building (apartment/store) (photograph)
Long Beach	278 Molino Avenue	-118.16	33.767	8	"High oven stove" with heavy mattress moved across floor.
Long Beach	220 E. Anaheim	-118.191	33.782	8	Damage to gable ends, etc, of 2-3 story brick buildings (photograph, Berner, 1981).
Long Beach	Corner of 7th and Redondo Streets	-118.152	33.775	8	Near total collapse of bank and Post Office buildings
Long Beach	Along Somerset Blvd	N/A	N/A	8.5	Partial/total collapse of many brick buildings
Long Beach	3027 East Sixth St.	-118.156	33.774	9	Objects in curio cabinet thrown into air and forward (Maher, 1933)
Long Beach	Morrison Apartments, 915 E. Ocean	-118.181	33.766	8.5	Partial collapse, 3-story brick apartment building
Long Beach	400 Pine Avenue	-118.193	33.798	8.5	Partial collapse of 2-story brick building (Berner, 1981).

Long Beach	Central fire station, 3rd and Pacific	-118.194	33.77	8.5	Partial collapse of 2-story brick firehouse (Berner, 1981)
Long Beach	345 Pine Avenue	-118.193	33.771	8	Damage to two-story Woolworth building (Berner, 1981)
Long Beach	Arnold's garage, Ocean and Termino	-118.146	33.76	8.5	Partial collapse of brick garage (Berner, 1981)
Long Beach	Partial collapse of auto agency	-118.19	33.784	8	Partial collapse (like that of other auto agencies with big display rooms) (Berner, 1981)
Long Beach	1253 E. 4th St.	-118.175	33.772	8	Partial collapse of two story brick building (Berner, 1981)
Long Beach	14th St. and Chestnut	-118.196	33.783	8	Collapse of walls at 3-story (brick) Seaside Hospital (Berner, 1981)
Long Beach	10th and Lindon	-118.186	33.779	8.5	Partial collapse of St. Mary's Hospital; "suffered more damage than other local hospitals; 3-story brick (Berner, 1981)
Long Beach	8th and Atlantic Ave.	-118.185	33.776	8.5	Partial collapse of large brick church (Brener, 1981)
Long Beach	St. Anthony's Catholic Church, 6th and Olive Ave.	-118.182	33.774	8.5	Collapse of steeples, gables, etc, large brick church (Brener, 1981)
Long Beach	1015 American Ave (now Long Beach Blvd)	-118.19	33.779	9	Total collapse of brick theater (Brener, 1981)
Long Beach	2157 Atlantic	-118.185	33.796	8	Damage to top of two-story brick building (Brener, 1981)
Long Beach	3rd and Cerritos Street	-118.178	33.77	8.5	Partial collapse of two -story masonry Ebel Club (Brener, 1981)
Long Beach	Broadway and Nieto	-118.133	33.765	8.5	Partial collapse, Lowell Elementary School, 2-3 story masonry (Brener, 1981)
Long Beach	Franklin Jr. High, 6th and Orange	-118.176	33.775	9	Near total collapse, three story brick school building (Brener, 1981)

Long Beach	17th and Walnut	-118.172	33.788	8	Partial collapse of large 2-story brick elementary school (Brener, 1981)
Long Beach	Blake Grocery, 2087 American Ave (now Long Beach Blvd)	-118.19	33.795	8.5	Partial collapse, two-story brick grocery (Brener, 1981)
Long Beach	1229 E. 4th St.	-118.176	33.772	8.5	Damage to top of well-constructed two-story brick building (Brener, 1981)
Long Beach	Orange and Anaheim St.	-118.176	33.783	8	Parial (front wall) collapse, 2-story brick building (Brener, 1981)
Long Beach	1100 American Ave (now Long Beach Blvd)	-118.189	33.781	8	Partial collapse, two-story building (Brener, 1981)
Los Alamitos	(Not specified)	-118.072	33.804	8.5	Modern brick building heavily damaged (photograph, California Institute of Technology archives)
Los Alamitos		-118.072	33.804	8	Two-story brick building heavily damaged (photograph, California Institute of Technology archives)
Los Angeles	Corner of Manchester and Alameda Street	-118.232	33.96	8	Near-total collapse of masonry garage (photograph)
Lynwood	Jefferson High School	-118.251	34.0096	9	Total collapse (photograph); infrastructure of 6 campus buildings destroyed.
Signal Hill	Security First National Bank (estimated location)	-118.211	33.93	8	Heavy damage to corner of 2-story brick bank (Photograph, Los Angeles Times)
	4 miles north of signal hill, Artesia Ave	-118.169	33.875	8.5	Concrete pavement buckled up 12" in one place, 6" in another; NS compression at Atlantic Ave. (crossing Artesia); garage on corner demolished; store front fell nearby
Seal Beach	8th near Central	-118.106	33.742	8.5	Cans flew off shelf, hit girl in face; 2-story brick building "fell like a house of cards" (Strawther, 2014)

Seal Beach	City Hall	-118.105	33.7419	7.5	Slightly damaged (Strawther, 2014)
Seal Beach	Jewel City Café	-118.106	33.7394	8	Part of café fell down (Strawther, 2014)
Seal Beach	School (now Mary Zoeter Elementary)	-118.101	33.743	8.5	Much of it demolished (Strawther, 2014)
Long Beach	Alamitos Bay Bridge	-118.113	33.7472	8.5	"Wrenched" (Strawther, 2014)
Long Beach	St. Anthony's Catholic Church (540 Olive Avenue)	-118.182	33.7736	8.5	Partial collapse, large, well-built church (Earthquake Pictures, 1933)
Long Beach	Redondo Avenue Bank of America	N/A	N/A	8.5	Near total collapse of brick bank building (Earthquake Pictures, 1933)
Long Beach	3rd and Pine	-118.192	33.7705	8.5	Wall collapse and other damage, two story brick rooming house (Earthquake Pictures, 1933)
Long Beach	City Hall (600 N. Alameda Street)	-118.22	33.8985	9	City Hall collapsed (grade 4 damage, 2-story masonry building, assume class B-C) (San Francisco Chronicle, 11 March; St. Louis University photograph: http://www.eas.slu.edu/eqc/eqc_photos/1933LBeq/)
Wilmington	Cherry near Willow	-118.168	33.8045	GF	Embankment gave way
Los Angeles	Harbor	-118.269	33.757	--	One killed, similar severity as San Pedro harbor (San Francisco Chronicle, 11 March)
Los Angeles	Florence and Compton Avenues	-118.248	33.9748	7	Partial collapse of 2-story brick building (grade 4 damage; assume class A), 3 killed (San Francisco Chronicle, 11 March)
Los Angeles	Broadway near First	-118.246	34.0536	6.5	Bricks fell from older buildings (Grade 2/3 damage; assume class A) (San Francisco Chronicle, 11 March)
Los Angeles	Central and Slauson	-118.246	33.989	7	Building collapsed (Grade 5 damage, assume class A) (San Francisco Chronicle, 11 March)
Los Angeles	94th and Western	-118.309	33.951	--	Water tank wrecked (San Francisco Chronicle, 11 March)

Los Angeles	Hall of Records, North Broadway	-118.243	34.0558	6.5	Bricks and coping fell (grade 2 damage, large stone masonry building; assume class BC) (San Francisco Chronicle, 11 March)
Los Angeles	Central and Slauson	-118.256	33.989	7	Auto agency collapsed (grade 5 damage; assume class A) (San Francisco Chronicle, 11 March)
Los Angeles	1039 Crocker	-118.251	34.034	7	Warehouse collapsed (grade 5 damage; assume class A) (San Francisco Chronicle, 11 March)
Los Angeles	8th and Central	-118.241	34.037	7	California bank collapsed (grade 5 damage; assume class A) (San Francisco Chronicle, 11 March)
Los Angeles	Central and 46th Street	-118.256	34.002	7	Warehouse collapsed (grade 5 damage; assume class A) (San Francisco Chronicle, 11 March)
Los Angeles	First and Alameda Streets	-118.238	34.049	6.5	"Severe" (grade 3/4) damage; assume class A (San Francisco Chronicle, 11 March)
Los Angeles	61st and Compton	-118.248	33.984	6.5	"Severe" (grade 3/4) damage (San Francisco Chronicle, 11 March)
Long Beach	Compton and Florence	-118.248	33.975	6.5	"Severe" (grade 3/4) damage (San Francisco Chronicle, 11 March)
Long Beach	Main Street	-117.868	33.748	7.5	Heavy damage (grade 3) to stores (New York Times, 12 March, pg 23)
Santa Ana	55th Street and Santa Fe	-118.23	33.993	7	Transportation building collapsed (Los Angeles Herald, 11 March)
Compton	4th Street and Sycamore			7	Grade 2/3 damage to downtown buildings (photograph)
Compton	South Oleander Avenue business district (111 South Oleander)	-118.229	33.896	8	Grade 3 damage to businesses (photograph)
Compton	Palmer's Garage, 215 N. Alameda Street	-118.22	33.895	8.5	Grade 5 damage (collapse) (photograph)
Huntington Beach	Business district	-118.001	33.6581	8.5	With one or two exceptions, every building in business district damaged from 50-100 percent (Los Angeles Times 12 March, pg 2)

Huntington Beach	Kenwood Hotel	N/A	N/A	8.5	"So damaged as to be practically uninhabitable" (Los Angeles Times 12 March 1933, pg 2)
Huntington Beach	Chamber of Commerce Bldg	N/A	N/A	8.5	"So damaged as to be practically uninhabitable" (Los Angeles Times 12 March 1933, pg 2)
Huntington Beach	Obarr Building	-118.002	33.6574	8	So damaged as to be practically uninhabitable (Los Angeles Times 12 March 1933, pg 2)
Huntington Beach	IOOF Hall	-117.999	33.6599	8	"So damaged as to be practically uninhabitable (Los Angeles Times 12 March 1933, pg 2)
Huntington Beach	Main street, several stores	-118.001	33.6581	8.5	"So damaged as to be practically uninhabitable (Los Angeles Times 12 March 1933, pg 2)
Huntington Beach	Telephone building	N/A	N/A	8.5	Concrete telephone building cracked top to bottom (Los Angeles Times 12 March 1933, pg 2)
Huntington Beach	City Hall	-117.999	33.6619	7.5	Severely cracked (Los Angeles Times 12 March 1933, pg 2)
Huntington Beach	High School	-118.003	33.6766	7	High school not damaged but water thrown from plunge onto floor (Los Angeles Times, 12 March 1933, pg 2)
Bolsa Chica Gun Club	Central Elementary School (now Dwyer Jr. High)	-118.004	33.6695	8	Grade 3-4 damage to Class B-C building (photograph)
Anaheim Bridge		-118.053	33.7041	7	Overall length of bridge reduced by about 9"; south end shifted north (Barrows, 1974).
2.5 miles south of Huntington Beach		-118.085	33.7313	9	"Signs of extreme shock along highway." (Maher, 1933)
Long Beach	Marti's Building, Pine Avenue	-117.967	33.6369	8.5	(Saint Louis University photograph)
Long Beach	Polytechnical High School	-118.192	33.771	9	Grade 3-4 damage to block masonry (class C) structure (Saint Louis University photograph)
Santa Ana	Furniture store, 4th and Spurgeon	-118.184	33.787	8.5	Grade 4 damage to brick building (class B) (Saint Louis University photograph)

Los Angeles	Compton Furniture Store	N/A	N/A	8	Damage to parapets, block-masonry (class B) structure (Saint Louis University photograph)
Bolsa Chica Gun Club		-118.304	33.937	7	Ground failure on road; no sign of damage to wood-frame structure (Barrows, 1974; USGS photograph)
Talbert Area		-118.053	33.7041	GF	(Barrows, 1974)
Westminster	South of Ocean and Westminster	-117.982	33.756	GF	Fissures/sand blows (Online Archives of California photograph)
Alamitos-Seal Beach		-118.111	33.752	GF	Sand blows (photograph)
Cabrillo Beach		-118.283	33.711	GF	Fissures/sand blows (Hillis, 1933)
Near Hog Island		N/A	N/A	GF	(Barrows, 1974)
1.4 miles SE of Huntington Beach pier		-117.982	33.645	GF	"volcanoes" (Barrows, 1974)
Between Newport and Laguna Beach		-117.851	33.584	GF	Cracks (Barrows, 1974)
Seal Beach	North end of beach	-118.113	33.742	GF	Ground failure on road (Barrows, 1974)

References

Barrows, A. G. A review of the geology and earthquake history of the Newport-Inglewood structural zone, Southern California, California Division of Mines and Geology Special Rep. **114**, 134 pp., Sacramento (1974).

Brener, L. (editor) Earthquake 33: A photographic history, Historical Society of Long Beach, 64 pp, Long Beach (1981).

Earthquake Pictures, (souvenir booklet), Green’s, Inc., Long Beach (1933).

Maher, T. J. Abstracts of reports received regarding the earthquake which occurred in Sourthern California on March 10, 1933," 56 pp. U.S. Coast and Geodetic Survey, San Francisco (1933).

Neumann, F. United States Earthquakes, U.S. Dept. of Commerce, Coast and Geodetic Survey Serial No. 579, 82 pp, Washington D.C. (1935).

Strawther, L. Seal Beach: A brief history, Arcadia Publishing, 128 pp, Charleston, South Carolina (2014).

Table S3. 1D Velocity Structure for High-Frequency Simulations

Thickness (km)	Vp (km/s)	Vs (km/s)	Density (g/cc)
0.01	1.50	0.425	2.00
0.02	1.60	0.538	2.05
0.07	1.80	0.65	2.10
0.10	2.00	0.80	2.20
0.30	3.10	1.40	2.30
0.50	3.70	1.80	2.45
0.50	4.20	2.10	2.575
1.00	4.75	2.40	2.60
1.00	5.10	2.75	2.62
1.50	5.40	3.00	2.65
2.00	5.80	3.35	2.70
2.00	6.20	3.50	2.72
8.00	6.35	3.60	2.75
13.00	6.80	3.80	3.00
-	7.80	4.50	3.30
